# A centrosome interactome provides insight into organelle assembly and reveals a non-duplication role for Plk4

**DOI:** 10.1038/ncomms12476

**Published:** 2016-08-25

**Authors:** Brian J. Galletta, Carey J. Fagerstrom, Todd A. Schoborg, Tiffany A. McLamarrah, John M. Ryniawec, Daniel W. Buster, Kevin C. Slep, Gregory C. Rogers, Nasser M. Rusan

**Affiliations:** 1 Cell Biology and Physiology Center, National Heart, Lung, and Blood Institute, National Institutes of Health, Bethesda, Maryland 20892, USA; 2 Department of Cellular and Molecular Medicine, University of Arizona Cancer Center, University of Arizona, Tucson, Arizona 85724, USA; 3 Department of Biology, University of North Carolina at Chapel Hill, Chapel Hill, North Carolina 27599, USA

## Abstract

The centrosome is the major microtubule-organizing centre of many cells, best known for its role in mitotic spindle organization. How the proteins of the centrosome are accurately assembled to carry out its many functions remains poorly understood. The non-membrane-bound nature of the centrosome dictates that protein–protein interactions drive its assembly and functions. To investigate this massive macromolecular organelle, we generated a ‘domain-level' centrosome interactome using direct protein–protein interaction data from a focused yeast two-hybrid screen. We then used biochemistry, cell biology and the model organism *Drosophila* to provide insight into the protein organization and kinase regulatory machinery required for centrosome assembly. Finally, we identified a novel role for Plk4, the master regulator of centriole duplication. We show that Plk4 phosphorylates Cep135 to properly position the essential centriole component Asterless. This interaction landscape affords a critical framework for research of normal and aberrant centrosomes.

## Abstract (web summary)

The centrosome is a large intracellular structure that serves as the microtubule-organising center, but how it is accurately assembled is not known. Here the authors generate a ‘domain-level' centrosome interactome and show that Plk4 positions the essential centriole component Asterless by phosphorylating Cep135.

## Graphical abstract

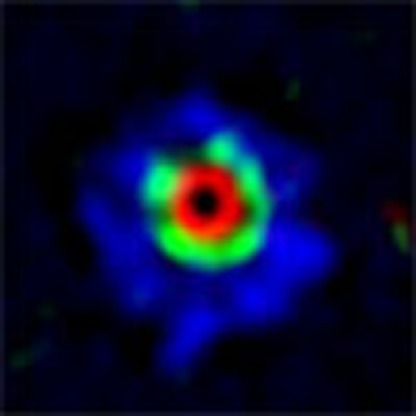

The centrosome, consisting of a pair of centrioles and pericentriolar material (PCM), is the major microtubule-organizing centre (MTOC) of many eukaryotic cells. Centrosome duplication and MTOC activity are tightly regulated to ensure that two centrosomes with optimal microtubule arrays are present at mitosis. Defects in the function of centrosomes and cilia, which are nucleated from specialized centrioles termed basal bodies, are implicated in a range of human diseases, including microcephaly, dwarfism and polycycstic kidney disease^1^,^2^. Understanding the molecular underpinning of the assembly, function and regulation of centrosomal proteins is a critical prerequisite for understanding the basis of human diseases.

Significant progress has been made towards understanding centrosome composition, duplication, maintenance and function. Mass spectrometry (MS) analysis of purified centrosomes identified hundreds of centrosome-associated proteins^3^,^4^,^5^. A much smaller set of proteins was identified as essential for centrosome duplication or activity through genetic analysis in model systems^6^ and high-throughput RNA interference screens in culture^7^,^8^,^9^. In contrast, there is a poor understanding of how centrosome proteins are assembled to form this structure and carry out its functions at the level of direct protein–protein interactions (PPIs). Given that the centrosome is a non-membrane-bound organelle, and it is effectively a gigantic protein complex, PPIs must drive its assembly. Furthermore, any modulation of its behaviour must be driven by alterations of PPIs among its constituents.

Identifying the direct PPIs among centrosome proteins has proven quite challenging. Genome-wide screens identified few interactions among centrosome proteins. The most successful of these, in *Caenorhabditis elegans*, only uncovered 10 interactions^10^. This low success rate of detecting PPIs is likely a result of limitations of the screens and is not representative of the true number of *in vivo* interactions. For example, in *Drosophila*, many more interactions have been identified in small-scale studies (Supplementary Table 3). Thus, new methods for identifying PPIs are critical for moving forward.

Another poorly understood aspect of centrosome regulation is the precise role of regulatory kinases. Only a few substrates have been identified for the master regulators of centriole duplication, Plk4, and centrosome maturation, Polo/Plk1 (refs 11, 12, 13, 14, 15, 16, 17, 18). Identifying novel substrates for these kinases, and other centrosome kinases such as Nek2, Aurora A and LK6, will uncover how they exert influence on centrosome assembly and function.

To rectify the significant deficit in our understanding of centrosome interactions, we generated a detailed centrosome interactome. Herein we report a directed yeast two-hybrid (Y2H) screen to identify the PPIs among 21 centrosome proteins. We use this information to gain an *in vivo* understanding of protein organization within the centrosome. We also demonstrate how the interactome can lead to the discovery of kinase substrates. Specifically, we uncovered a centriole duplication-independent role for Plk4, showing that it phosphorylates the centriole protein Cep135 to regulate its interaction with Asterless (Asl) and influence the radial positioning of Asl on centrioles.

In summary, we have exposed the highly interconnected nature of the centrosome, identifying 189 ‘strong' interactions and increasing the number of known PPIs tenfold. Future studies should specifically benefit from the PPIs we report, and generally benefit from the Y2H screening approach we employ.

## Results

### The extensive interaction potential of centrosome proteins

**Figure 1:**
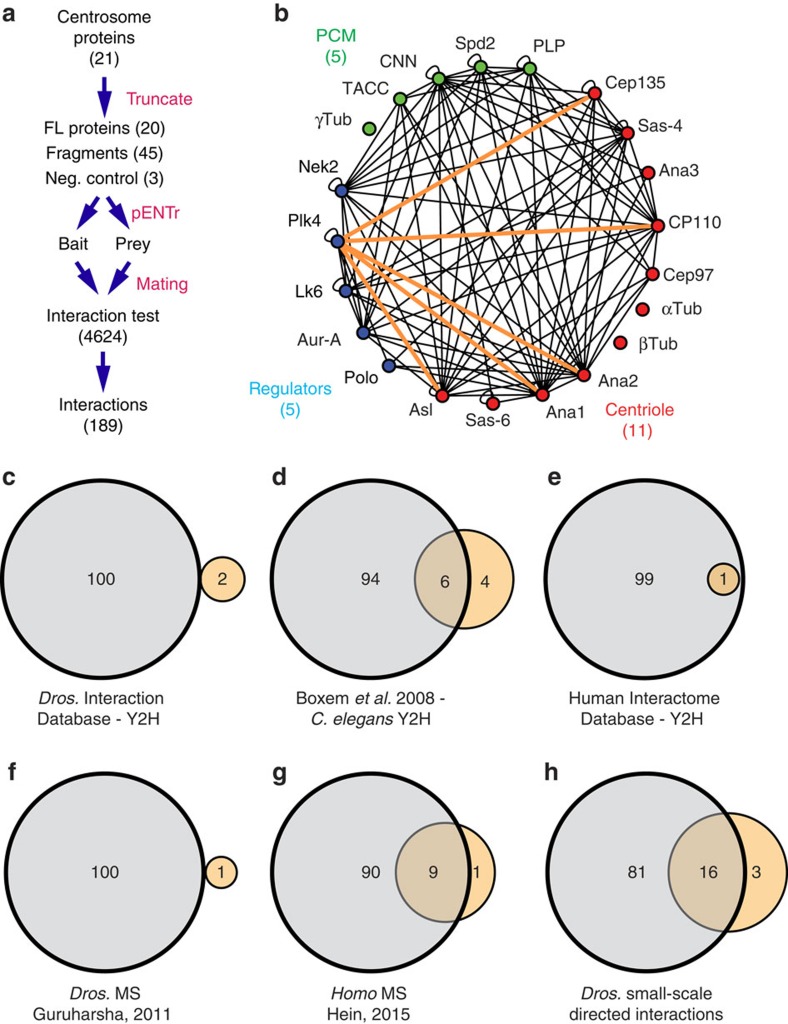
Y2H screen to determine the centrosome interactome. (**a**) Workflow of the centrosome array-based Y2H screen. (**b**) Summary of all interactions among the 21 centrosome proteins. Interaction details are summarized in Supplementary Material. Orange lines highlight the interactions made by Plk4 with proteins of the centriole. (**c**–**h**) Venn diagrams showing the overlap of the PPIs identified in this study (beige) and other studies (grey; Supplementary Table 3). (**c**) Overlap with Y2H data in the *Drosophila* Interaction Database^24^. (**d**) Overlap with *C. elegans* Y2H data from Boxem *et al*.^10^. (**e**) Overlap with Y2H data in the Human Interactome Database^25^,^26^. (**f**) Overlap with a high-throughput pull-down/mass spectrometry study in *Drosophila*^27^. (**g**) Overlap with high-throughput pull-down/mass spectrometry study in HeLa cells^28^. (**h**) Overlap with direct PPIs identified in small-scale studies in *Drosophila*.

To determine the potential interaction landscape of centrosome proteins, we performed a directed, array-based Y2H screen. We selected 21 conserved *Drosophila* centrosome proteins (11 centriole, 5 PCM and 5 regulatory kinases; Supplementary Fig. 1a; Supplementary Table 1; refs 6, 19). We developed a streamlined workflow for our mating-based screen (Fig. 1a), which we previously described in detail^20^. In addition to identifying interactions between full-length (FL) proteins, we aimed to define smaller regions within each protein sufficient for direct PPI. We used a proteomic approach to subdivide proteins into fragments of 200–600 amino acids. To maximize the possibility of obtaining functional fragments, great care was taken to maintain known, or predicted, motifs and secondary structures (Methods). Out of 21, 16 proteins were subdivided into 45 smaller polypeptides (Supplementary Table 2). The sequence encoding each protein and protein fragment was fused to yeast GAL4 AD (bait) and BD (prey), and all 4,624 bait/prey combinations were tested using a three-tiered, variable stringency reporter system (Supplementary Fig. 1b,c; Supplementary Data 1), which provided information about weak and strong interactions. Empty vectors were included to identify proteins that auto-activate the reporters in isolation. In addition, coiled-coil regions from non-centrosomal proteins, Syntaxin A and CG42673, were included as negative controls. At the lowest stringency we found 510 interactions. At moderate stringency we found 292 interactions. At the highest stringency we found 189 interactions (Fig. 1b, Supplementary Data 2 and Supplementary Data 3). For the remainder of this study we only consider the 189 interactions seen at the highest stringency (Fig. 1b), but stress that many bona fide interactions may only be found at lower stringencies. We report interactions at all stringencies and data for independent replicates online (Supplementary Data 1).

Previous large-scale and genome-wide screens have not provided significant insight into direct PPIs of centrosome proteins. For example, large-scale Y2H screens in *Drosophila* (*Drosophila* Interaction Database^21^,^22^,^23^,^24^) only revealed two interactions among the 21 proteins in our screen (Fig. 1c and Supplementary Table 3a). A Y2H-based screen in *C. elegans*^10^ identified 10 direct interactions among centrosome proteins in our screen, 6 of which we also identified using the orthologous *Drosophila* proteins (Fig. 1d and Supplementary Table 3b). Finally, two large-scale Y2H-based interactomes (Human Interactome Database; interactome.dfci.harvard.edu/H_sapiens/ (refs 25, 26)) only found one interaction among the human orthologues of centrosome proteins in our screen (Fig. 1e and Supplementary Table 3c), which we also identified. In contrast to these genome-wide approaches, our interactome reveals the power of a more focused approach, where precise protein truncations, attention to every yeast transformation and mating pair, and careful analysis of each interaction, resulted in a dramatic increase in detected PPIs.

To compare our Y2H approach to other approaches, we examined large-scale affinity purification/MS (AP-MS) studies. In *Drosophila*, only one pair of proteins was co-purified in an AP-MS screen (Fig. 1f and Supplementary Table 3d (ref. 27)). A screen in HeLa cells identified 10 pairs of proteins included in our screen that were co-purified, 9 of which the *Drosophila* orthologues make direct PPIs (Fig. 1g and Supplementary Table 3e (ref. 28)). An emerging technology to study the local protein environment in its cellular context is proximity biotin labelling. Two studies used this technique to examine proteins included in our screen^29^,^30^. Similar to AP-MS screens, this technique does not reveal direct PPIs, and, similar to all techniques, including our Y2H screen, does not inform where, when or how long the proteins were in proximity. However, in combination with other methods, proximity labelling will be critical for investigating centrosome protein assembly. In the discussion, we consider an example of the overlap between our study and proximity labelling studies as it relates to CPAP/Sas-4.

In contrast to all the high-throughput approaches, primary studies on individual centrosome proteins have yielded several important interactions. A survey of the literature for direct PPIs identified in small-scale studies in *Drosophila* found 19 PPIs (to the best of our knowledge), 16 of which were identified in our screen (Fig. 1h and Supplementary Table 3f). This demonstrates that our approach is successful in uncovering bona fide interactions previously shown to be critical for centrosome function. It also suggests that the new PPIs that we identified will prove to be biologically relevant. Finally, our comparison with published data indicates that high-throughput PPI screens have not substantially revealed the interaction landscape of the centrosome and, therefore, are not optimal for investigating centrosome PPIs, and possibly not optimal for other complex protein networks.

### High connectivity and high confidence interactions

We identified several ‘high-connectivity' regions of centrosome proteins. While these regions may serve as regulatory nodes or scaffolds, the number of interactions at these sites makes them the most difficult to functionally dissect. Examples of these high-connectivity regions are the N-terminus of Cnn (24 interactions), the C-terminus of Asl (21 interactions) and the C-terminus of Ana2 (23 interactions). The complexity of the PPIs formed by these regions suggests that they may have roles beyond the singular roles ascribed to them in the literature.

One approach to prioritizing the effort of centrosome research is to identify regions that display few interactions, assuming that fewer interactions indicate high specificity. In addition, these interactions would be the easiest to specifically disrupt and study. We developed a scoring system to identify these interactions and term them ‘high confidence interactions' (HCIs; Methods; Table 1). Of the 37 HCIs (top 20% of all interactions), 14 HCIs are formed by FL proteins. This was predictable, given so few PPIs formed with FL proteins. Thirteen HCIs were previously identified in at least one system (Table 1), including the interactions between the master centriole duplication kinase Plk4 and its centriole-anchor Asl (refs 31, 32). The remaining 24 HCIs are yet to be reported and therefore possess the greatest potential for future studies.

Of the 15 proteins tested as FL and fragments, only 6 showed interactions as FL. In contrast, at least one fragment from all 15 formed one or more PPI. This suggests that intermolecular interactions might be masked by intramolecular interactions, and that this might be a common regulatory mechanism employed at centrosomes (Discussion). Although we highlight one method of prioritizing the interactome based on the least promiscuous polypeptides, other prioritization methods could be employed to identify HCIs.

### The complexity of centriole–protein interactions

**Figure 2:**
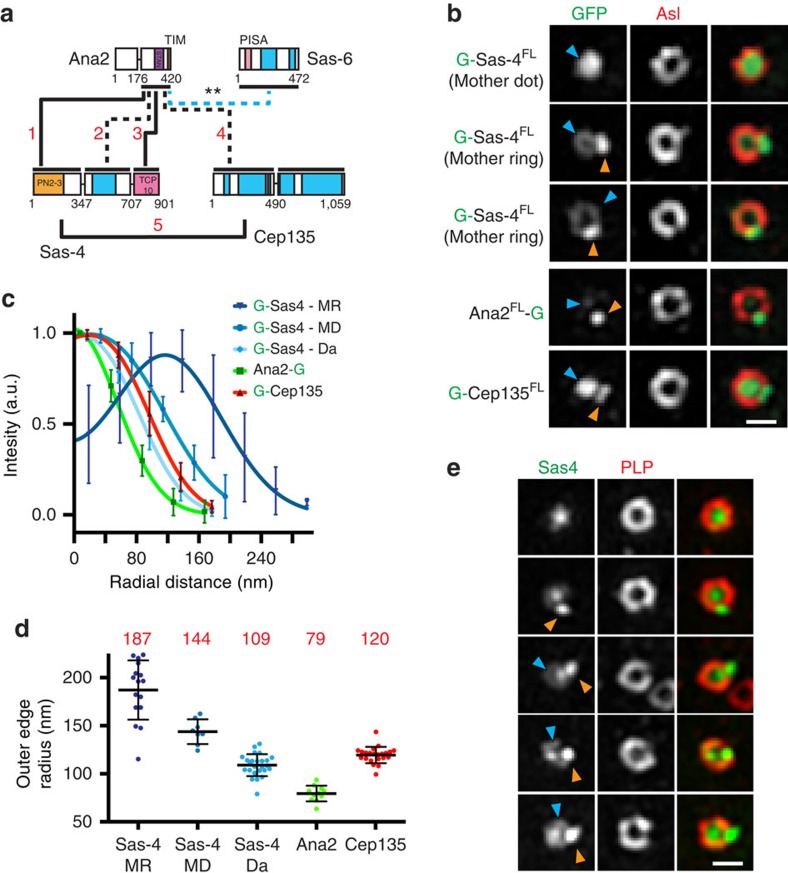
Centriole–protein interactions reveal dynamic and stable components. (**a**) Interactions (red numbers) among core centriole proteins. Black numbers are amino acids, blue regions are predicted coiled-coils, horizontal lines indicate the locations where proteins were subdivided, dark black lines are interactions scoring 2 or 3, dashed lines scored 1 and blue dotted line indicates a phosphorylation-dependent interaction (Fig. 6d)^16^,^17^. (**b**) SIM of centrioles from S2 cells expressing GFP::Sas-4. Sas-4 presents as dots or rings in mothers and only as dots in daughters. Ana2 is always a dot. Cep135^N-term^ forms small rings. Blue arrowheads, mother. Orange arrowheads, daughter. (**c**) Averaged intensities of linescans across centrioles aligned from the centriole centre and moving out. Lines are fits to average data. Error is ±s.d. Ns are centrioles, Sas-4 (MR, mother ring)=16, Sas-4 (MD, mother dot)=8, Sas-4 (Da, daughter)=25, Ana2=11, Cep135=22 from two experiments. (**d**) Outer edge measurements showing the mean (red number) of individual fits of data in **c** ±s.d. (**e**) SIM of centrioles in wing discs from transgenic flies expressing Sas-4::GFP. Similar to in S2 cells Sas-4 occupies a variety of positions within centrioles in the wing disc. Scale bars, 400 nm.

A set of four conserved core proteins, Sas-6, Ana2, Cep135 and Sas-4, is required to build the centriole^19^. Our screen identified five interactions among three of these centriole proteins (Fig. 2a and Supplementary Fig. 2a), adding to the limited known interaction data. While current models of the centriole architecture predict heavy reliance on stable PPIs, the interactions we identified do not support a simple model where all PPIs are represented in the final static centriole structure. Our data are more consistent with a dynamic model where some transient interactions are required during centriole assembly, while other interactions are used to anchor a protein in its final position.

To illustrate the varied localization of centrosome proteins, which would support dynamic and/or transient interactions, we focused on Sas-4, which forms four of five intercentriolar interactions identified. Three PPIs occur with Ana2^C-term^, which localizes as a dot on mother and daughter centrioles with an outer edge of 79.4±8 nm (Fig. 2b–d). Another interaction occurs with Cep135^N-term^, which localizes as a ring on both mothers and daughters with an outer edge of 120±9 nm (Fig. 2b–d). Thus, Sas-4 can interact with a central (Ana2) and a peripheral (Cep135) centriole component. This suggests that Sas-4 is present as subpopulations, or that Sas-4 localization changes, possibly engaging different PPIs for different tasks. This is supported by previous structured illumination microscopy (SIM) studies that localize Sas-4/CPAP to different regions of the centriole. *Drosophila* Sas-4 was localized as a ring around the centriole with a 100–150 nm radius^33^,^34^, while its mammalian orthologue CPAP was localized simultaneously to a ring and a dot^35^. We revisited the localization of Sas-4 by SIM and find it extremely variable (Fig. 2b–d). GFP::Sas-4 in cultured *Drosophila* cells formed a dot on daughter centrioles (outer edge radius=109±11 nm) and a larger dot (outer edge radius=144±13 nm) or a ring (outer edge radius=187±31 nm) on mothers. We also document a nearly identical variety of localizations in wing discs of *Drosophila* larvae (Fig. 2e).

Combining the direct PPI information with the highly variable Sas-4 localizations presents a testable model whereby Sas-4 interacts with Ana2^C-term^ (Fig. 2a, interactions 1,2,3; Supplementary Fig. 2b,b′, co-immunoprecipitation (IP)) early in centriole construction, followed by interaction with Cep135^N-term^ in later stages of construction (Fig. 2a, interaction 5, Supplementary Fig. 2c, co-IP). Finally, the largest mother-centriole Sas-4 rings at 187 nm may represent an additional role for Sas-4, such as its proposed PCM scaffold/recruitment role^36^. Consistent with such a role, Sas-4 interacted with several PCM proteins (Fig. 1b, Supplementary Data 2 and Supplementary Data 3). Thus, our interactome can be used to parse apart and uncover new roles for multifunctional proteins, such as Sas-4.

In addition to core centriole proteins (Fig. 2a), our interactome provides context and insight into less well-studied molecules within the centriole. For example, Ana1, an essential centriole protein, forms 11 interactions with core centriole proteins (Supplementary Fig. 3a,b). Three interactions are HCIs, two of which form between Ana1^N-term^ and Cep135^N-term^, recently implicated together in an early centriole assembly^37^. This strongly validates our Y2H screen and encourages future studies on the remaining Ana1 interactions. One exciting interaction in particular is Ana1 with Sas-6, as it represents the only interaction we identified between Sas-6 and another centriole protein. We confirmed this interaction using co-IP (Supplementary Fig. 3c). These data also encourage screening any centriole protein identified in the future against our Y2H library, as our approach offers a quick view of a protein's interaction potential.

### Oligomerization is a hallmark of centrosome proteins

**Figure 3:**
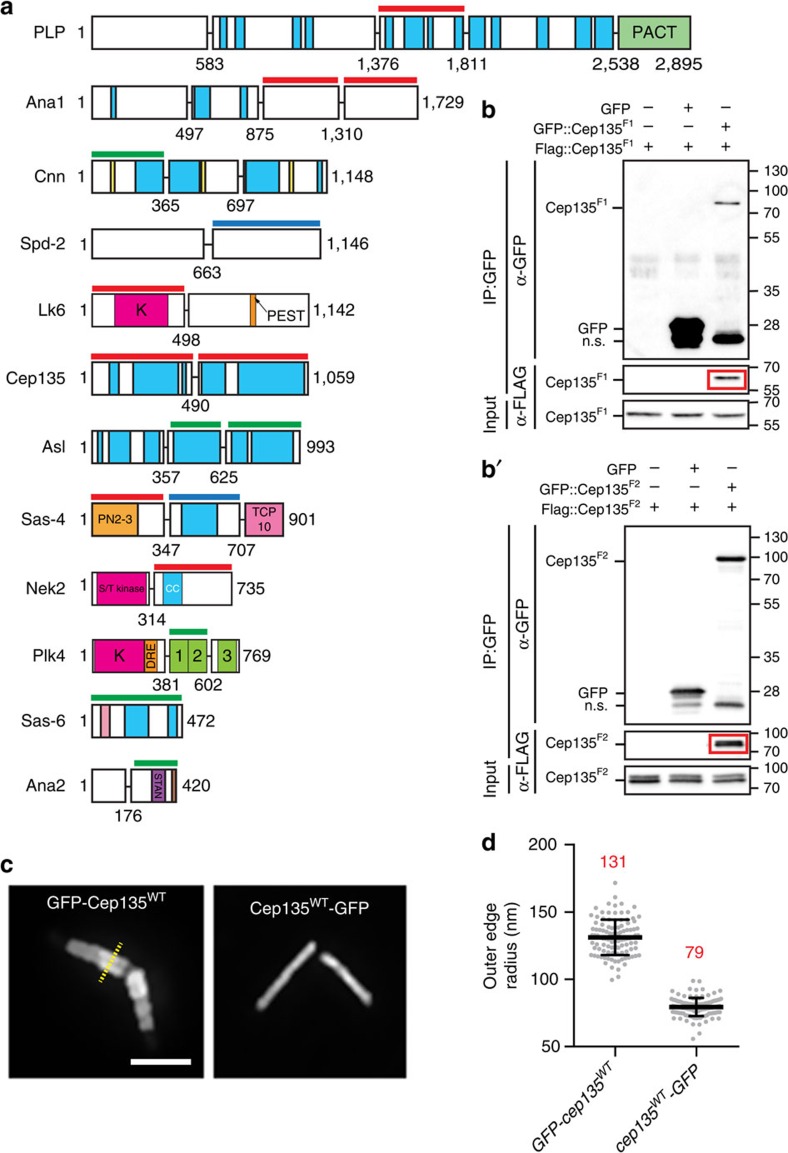
Multimerization is a common feature of centrosome proteins. (**a**) Schematics (as in Fig. 2a) of all of the proteins in the screen that contained a region able to self-associate. Red lines indicate fragments with novel interactions. Blue lines indicate fragments where the interaction is known in a different experimental system or where the region of interaction in *Drosophila* has been refined by our screen. Green lines indicate previously described interactions. Numbers are amino acids. Corresponding Y2H data are in Supplementary Fig. 4. (**b**,**b′**) Co-IPs showing the Cep135^N-term^ and Cep135^C-term^ self-associations. Red box highlights protein in the IP. Sizes are in kD. (**c**) Cep135 adopts an extended conformation in centrioles. Sagittal views of centrioles from spermatocytes in transgenic flies expressing Cep135 tagged with GFP at the N- and C-termini. All measurements of spermatocyte centrioles were taken with a line scan perpendicular to the long axis of centrioles (representative linescan, yellow). (**d**) Outer edge measurements of centrioles from spermatocytes in transgenic animal testes. Mean (red number) ±s.d. Ns are centrioles—GFP::Cep135=109, Cep135::GFP=117, pooled from three experiments. Scale bar, 1 μm.

A striking finding in our data is the high frequency of protein self-association—16 protein fragments (nine known and seven novel) across 12 proteins (Fig. 3a and Supplementary Fig. 4). This suggests that higher-order oligomerization is a ubiquitous mechanism used to construct and regulate centrosomes, as previously demonstrated for Ana2, Sas-6, Asl, Cnn and Plk4 (refs 12, 38, 39, 40). As a new example, we explore the Cep135^N-term^ and Cep135^C-term^ self-association, which we verified using co-IP (Fig. 3b,b′). The ability of Cep135 to interact with itself at both termini raises the possibility that Cep135 might form an extended dimer or multimer. To test this model we examined the ‘giant' centrioles in the spermatocytes of testes of *Drosophila* expressing N- or C-terminally GFP (green fluorescent protein)-tagged versions of Cep135 (Fig. 3c). Cep135^N-term^ was resolved as two lines along the entire centriole length with an outer edge radius of 131±13 nm (Fig. 3d). In contrast, Cep135^C-term^ is diffraction-limited and appears as a single line in the middle of the centriole (Fig. 3c) with an outer edge radius of 79±7 nm (Fig. 3d). This indicates that Cep135 adopts an extended conformation with its C-terminus in the middle of the centriole, and its N-terminus extended radially outwards. This molecular architecture is likely reinforced by the lateral intermolecular interactions along the length of Cep135. More broadly, oliogomerization of centrosome proteins affords a powerful mechanism to control all aspects of centrosome biogenesis, including duplication, maturation and MTOC function. Investigating the novel self-associations of Ana1, PLP, Sas-4 and Cep135 should be a priority.

### Insights into the nature of PCM

**Figure 4:**
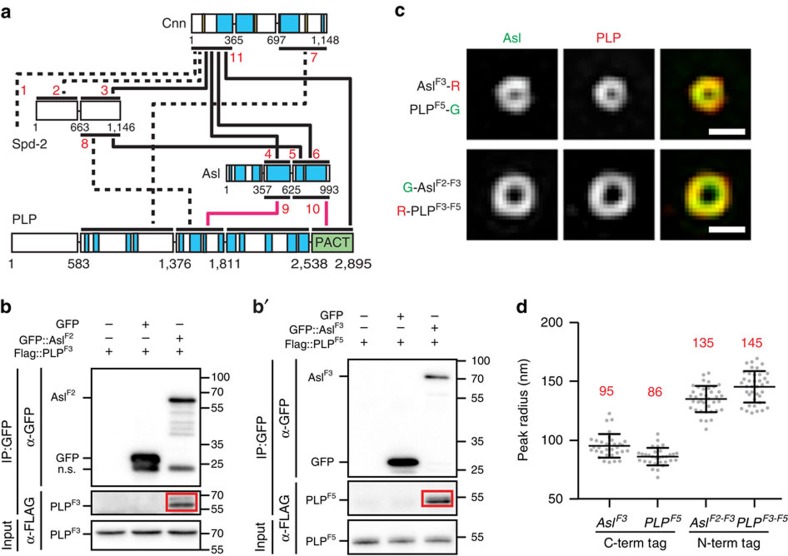
PCM protein interactions predict position and function. (**a**) PPIs among PCM proteins. Numbers refer to Y2H data in Supplementary Fig. 5a. Pink lines highlight interactions confirmed in **b**,**b**′. Schematics as in Fig. 2a. (**b**,**b′**) Co-IP confirmation of Asl^F2^ (aa 358–625)/Plp^F3^ (aa 1,377–1,811) and Asl^F3^ (aa 626–993)/Plp^F5^ (aa 2,539–2,895) interactions (interactions 9 and 10 from **a**). Red box highlights co-IP'ed protein. Sizes are in kD. (**c**) SIM of representative centrioles from S2 cells expressing the tagged Asl and Plp fragments diagrammed in Supplementary Fig. 5b. Both of the regions of the two proteins that interact are in close proximity to each other on centrioles. (**d**) Radius of the N- and C-termini of Asl and PLP on centrioles (as in **c**). Peak radius measurements showing the mean (red number)±s.d., Ns are centrioles—Asl^F3^=32, PLP^F5^=31, Asl^F2–F3^=38, PLP^F3–F5^=38, pooled from two experiments. Scale bars, 400 nm.

The nature of the PCM is one of the most profound questions in centrosome biology. We focused on the interactions among four proteins thought to scaffold the PCM (Asl, PLP, Cnn and Spd2) and uncovered extensive interactions among them (Fig. 4a and Supplementary Fig. 5a). As it relates to PCM assembly, our interactome supports two distinct, but not mutually exclusive, models. Model 1: PCM proteins form a large number of multivalent interactions (Fig. 4a, Supplementary Data 2, Supplementary Fig. 5a) that can be leveraged to assemble a membraneless organelle. Our data are therefore consistent with the hypothesis that centrosomes represent phase-separated droplets^41^ within the cytoplasm that rely on extensive PPIs. In fact, we identified fragments within each PCM protein that form many interactions, providing the multivalent interaction landscape required for phase separation. These fragments include Cnn^F1^ (24 interactions), Spd-2^F2^ (11 interactions), PLP^F3^ (12 interactions), Asl^F2^ (10 interactions) and Asl^F3^ (21 interactions). One future approach to test this model is to modulate the number of PCM PPIs and assess the efficiency of organelle assembly *in vitro* and *in vivo*.

Model 2: PCM is anchored to the centriole wall via bridging proteins. Our interactome reveals an extensive binding between Spd2 and Cnn (Fig. 4a and Supplementary Fig. 5a), the major PCM scaffolds for γ-Tubulin ring complexes. Moreover, Spd2 and Cnn form strong interactions with Asl and PLP (Fig. 4a and Supplementary Fig. 5a), which localize close to the centriole wall^34^,^42^. Thus, our interactome has potentially defined the majority of the direct PPIs that link the PCM with centrioles, a requisite for centrosome maturation and function. Specifically, we suggest that Asl–Cnn (Fig. 4a, Interactions #4, 6) and PLP–Cnn (Fig. 4a, int. #7, 11 (ref. 43)) are critical for PCM anchorage. Thus, our interactome can challenge existing paradigms while providing a critical framework with which to build new models. These data could also aid efforts to identify the minimum components required for PCM function, providing a framework to refocus efforts for biochemical and *in vitro* reconstitution experiments using protein fragments that serve as interaction nodes.

### Spatial localization of bridging proteins

The model for Asl and PLP serving as PCM-bridging/anchoring proteins stems, in part, from their conformation; both extend outwards from the centriole wall^34^,^42^. Asl and PLP do not rely on one another for localization; however, a reciprocal functional dependency remains unexplored. We identified two interactions between Asl and PLP (Fig. 4a, interactions # 9, 10), confirmed using co-IP (Fig. 4b,b′). These interactions predict similar radial positioning of the interacting regions. To test this, we generated N- and C-terminal fluorescent tags of Asl and PLP protein truncations (Supplementary Fig. 5b). Introducing combinations of these transgenes into cultured S2 cells, we show that the C-termini of Asl and PLP bridged by interaction 10 reside at a radius (peak, Supplementary Fig. 2d′) of 95±10 and 86±7 nm, respectively (Fig. 4c,d), placing the C-terminal interacting regions in close proximity. Similarly, the middle region of Asl and PLP (Supplementary Fig. 5b), bridged by interaction #9, resides at 135±11 and 145±13 nm (Fig. 4c,d), again placing the interacting regions in close proximity. Interestingly, the position of the N-termini of these truncations is almost identical to the N-termini of the FL proteins measured at 135±11 nm for Asl and 146±11 nm for PLP^34^. This indicates that the first 357 residues of Asl and 1,376 residues of PLP do not add length to the protein, possibly because they adopt folded conformations. Thus, combining SIM localization with our PPI data is a powerful approach to understand centrosome protein assembly *in vivo*. Furthermore, combining mutations generated to disrupt individual interactions, as we have previously outlined^20^, with SIM localization will be critical for functional analysis of all centrosome proteins. Our interactome provides a necessary prerequisite for this research approach, particularly for generating mutant alleles.

### Uncovering kinase substrates and regulated interactions

**Figure 5:**
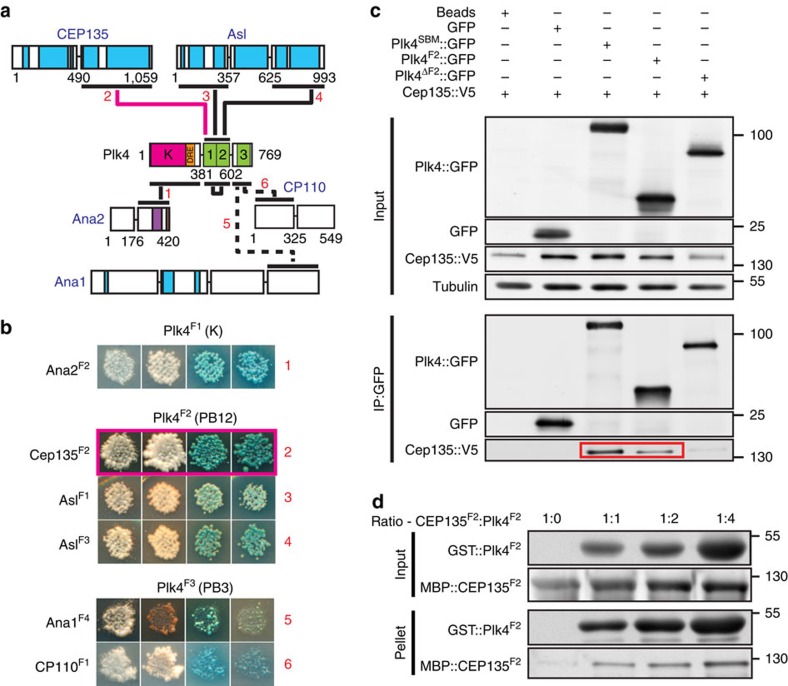
Plk4 interacts with Cep135. (**a**) All Plk4 interactions identified in screen (red numbers refer to **b**). Pink line highlights the interaction of Cep135 and Plk4. Schematics as in Fig. 2a. (**b**) Results of Y2H interactions with Plk4. Red numbers refer to interactions indicated in **a**. Y2H data are arranged as in Supplementary Fig. 1c. Pink box highlights the interaction of Plk4 with Cep135. (**c**) Plk4^F2^ (aa 382–602), containing Polo Boxes 1 and 2, is necessary and sufficient for interaction with Cep135. GFP was IP'ed from S2 cells expressing the indicated constructs. Plk4^SBM^ contains a mutation in the Slimb-binding domain that causes stabilization of Plk4 (ref. 44). Plk4^ΔF2^ is missing residues 382–602 (Polo Boxes 1–2). Red box highlights that Cep135 is Co-IP'ed by Plk4^SBM^ and Plk4^F2^. Sizes are in kDa. (**d**) Recombinant Plk4^F2^ and Cep135^F2^ (aa 491–1,059) interact *in vitro*. Recombinant Plk4^F2^ and Cep135^F2^ were mixed at the indicated ratios and GST was pulled down. Increasing amounts of Plk4^F2^ pull down increasing amounts of Cep135.

The regulation of centrosome number and activity is critical for proper cellular function. For this reason, we included several kinases known to regulate centrosome biology in our screen (Supplementary Fig. 1a). To illustrate how the interactome can uncover regulatory mechanisms, we hypothesized that kinase-binding partners are also substrates. We explored this by investigating the PPIs identified with Plk4, the master regulator of centriole duplication (Figs 1b and 5a,b). We found that Ana1, Ana2, Asl, CP110, Cep135 and Plk4 itself are direct binding partners of Plk4. Four of these were HCIs with Plk4 (Table 1), three of which are known Plk4 substrates—Ana2, Asl and Plk4 itself^16^,^31^,^32^,^44^,^45^,^46^,^47^. The fourth, Cep135, is a previously unreported interaction.

**Figure 6:**
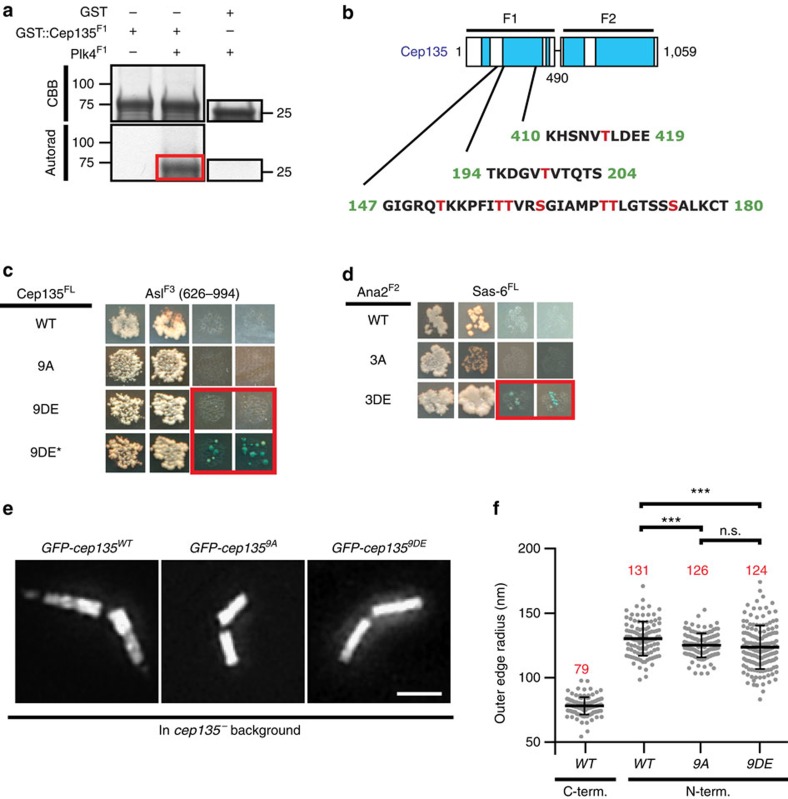
Plk4 phosphorylates Cep135 and regulates its interactions. (**a**) Plk4 phosphorylates Cep135^1–490^ (red box) in an *in vitro* kinase assay. Coomassie brilliant blue (CBB) gel (top) and autorad (bottom); GST used as negative control (right). (**b**) Schematic of Cep135 indicating the phosphorylated residues (red) identified by *in vitro* kinase assay with Plk4. (**c**) Phosphomimetic Cep135^FL-9DE^ gains interaction with Asl^F3^ (red box), while Cep135^FL-WT^ and Cep135^FL-9A^ do not. Images are representative of four independent experiments. Asterisk indicates 10-day growth. (**d**) Phosphomimetic (3DE) Ana2^F2^ interacts with Sas-6^FL^ (blue box), but wild type (WT) and unphosphorylatable (3A) Ana2^F2^ do not. Y2H data are arranged as in Supplementary Fig. 1c. (**e**) Images of spermatocyte centrioles from transgenic animals expressing the indicated constructs in the *cep135* mutant background. GFP-tagged WT, unphosphorylatable (9A) and phosphomimetic (9DE) Cep135 localize to centrioles in the spermatocytes of transgenic flies. (**f**) Outer edge radii of Cep135 on spermatocyte centrioles. Bars indicate the mean (red numbers)±s.d. Numbers of centrioles measured—WT^C-term^ *N*=117 (same data as in Fig. 3d), WT^N-term^ *N*=109 (same data as in Fig. 3d), 9AN=124, 9DE *N*=154. Comparisons made by unpaired *t*-tests, with Welch's corrections when appropriate. ***P≤0.001, n.s., not significant. Scale bars, 1 μm.

We first verified the Plk4 and Cep135 interaction using co-IPs from S2 cells (Fig. 5c) showing that the Polo Boxes 1–2 cassette of Plk4 is both necessary and sufficient to bind Cep135. We then demonstrated, using recombinant, purified proteins, that Polo Boxes 1–2 and the C-terminal half of Cep135 interact *in vitro* (Fig. 5d). Finally, we performed *in vitro* kinase assays with recombinant Plk4^1–317^, revealing that Cep135 is a Plk4 substrate (Fig. 6a). Therefore, our interactome can indeed predict protein kinase substrates. Future analysis should be extended to the four other kinases and their binding partners, beginning with the eight HCIs (Table 1).

### Plk4 phosphorylates Cep135 to position Asl

Although we discovered Cep135 to be a Plk4 substrate *in vitro*, we aimed to investigate its physiological relevance. We used MS to identify nine phosophorylated residues in Cep135^N-term^ (Fig. 6b and Supplementary Table 4). To determine how Cep135 function might be regulated by phosphorylation, we generated a FL phosphomimetic Cep135 mutant (Cep135^9DE^) and then screened it against the entire centrosome Y2H library. This uncovered a single new interaction between Cep135^9DE^ and Asl^C-term^ (Fig. 6c). To confirm that phospho-regulated interactions could be studied with our Y2H screening method using phophomimetics, we demonstrated that a phosphomimetic mutant of the C-terminus of Ana2 (3DE), but not WT or an alanine mutant (3A), can bind Sas-6, as was previously shown using co-IP (Fig. 6d) (refs 16, 17). Interestingly, our initial screen identified an interaction between Cep135^N-term^ and Asl^C-term^ fragments, but not between Cep135^FL^ and Asl^C-term^. This suggests that, in the context of FL Cep135, the Cep135^N-term^ interaction with Asl^C-term^ is inhibited and that phosphorylation relieves this inhibition.

**Figure 7:**
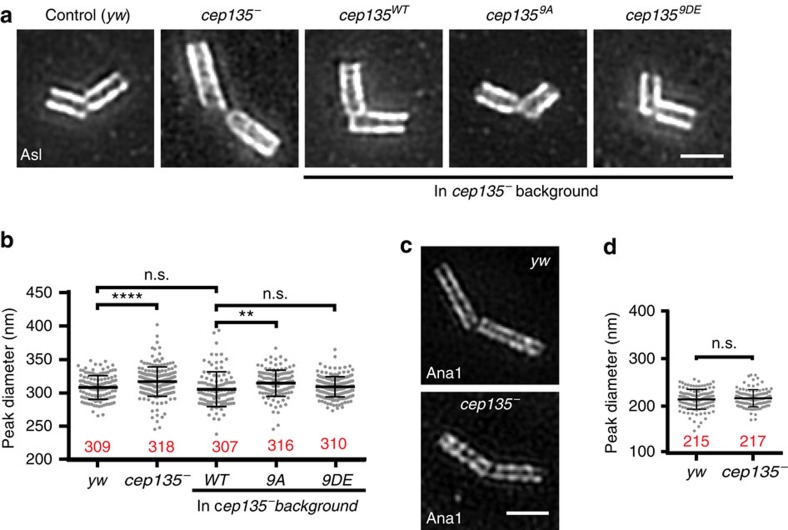
Phosphorylation of Cep135 is critical for positioning Asl on centrioles. (**a**) SIM of Asl in spermatocyte centrioles from testes of the indicated genotypes. (**b**) The peak diameter of Asl in *cep135* mutant centrioles is rescued by expression of Cep135^FL-WT^ and Cep135^FL-9DE^, but not Cep135^FL-9A^. Bars indicate the mean (red numbers) ±s.d.; Ns are centrioles—*yw=*138, *cep135*=199, *WT*=112, *9A*=132, *9DE*=192; pooled from three experiments. (**c**) Ana1::tdtomato direct fluorescence in control (yw) and cep135 mutant centrioles. Loss of Cep135 does not affect Ana1 positioning. (**d**) Peak diameter measurements of Ana1-labelled spermatocyte centrioles. Ana1 position is unaffected by the absence of Cep135. Bars indicate the mean (red numbers)±s.d. Number of centrioles measured—yw=124, cep135=112. Comparisons made by unpaired *t*-tests, with Welch's corrections when appropriate. ****P<0.0001, **P≤0.01, n.s., not significant. Scale bars, 1 μm.

To determine whether phosphorylation of Cep135 on these residues was critical for cellular and tissue function, we generated flies expressing Cep135^WT^, Cep135^9A^ or Cep135^9DE^ and tested whether these could rescue the known radial displacement of Asl in *cep135* mutant spermatocytes^48^ (Fig. 6e). The following experiments were performed using the ‘giant' centrioles of *Drosophila* spermatocytes. The radial positions of the Cep135^9A^ (251±18 nm) and Cep135^9DE^ (248±33 nm) proteins themselves (as measured with an N-terminal GFP tag) were similar, although both were slightly narrower than Cep135^WT^ (261±26 nm; Fig. 6e,f). We confirmed the published result showing that Asl is positioned further away from the centre of the centriole in *cep135* mutants (Fig. 7a,b). Consistent with Cep135 phosphorylation having a role in positioning Asl, the unphosphorylatable Cep135^9A^ does not rescue the radial position of Asl, while Cep135^WT^ and the phosphomimetic Cep135^9DE^ do (Fig. 7a,b). Importantly, loss of Cep135 does not disrupt the position of Ana1 (Fig. 7c,d), indicating that there is not a general defect in positioning centriole proteins in *cep135* mutants and the effect is specific to Asl.

**Figure 8:**
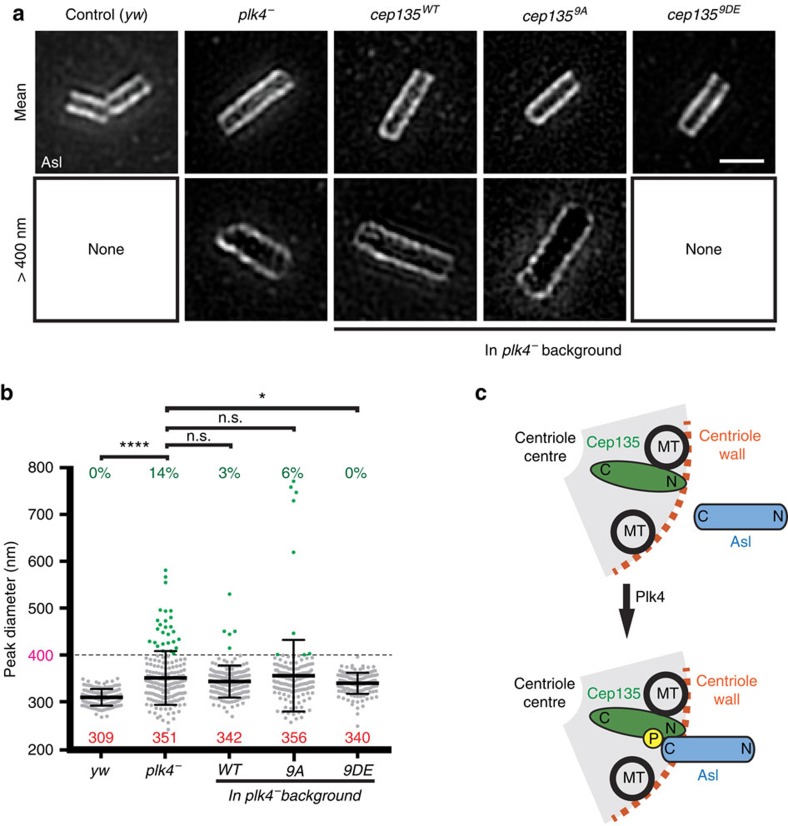
Plk4 is critical for Asl positioning on centrioles via Cep135 phosphorylation. (**a**) SIM of Asl immunofluorescence in spermatocyte centrioles. Representative centrioles at approximately the mean Asl diameter (top) and with Asl diameter >400 nm (>5 s.d.'s, bottom) of indicated genotypes; yw and Cep135^FL-9DE^ (in *plk4* background) show no centrioles >400 nm. (**b**) *plk4* mutant centrioles show great expansion of Asl peak diameter, which is partially rescued by Cep135^FL-9DE^. Bars indicate the mean (red numbers) ±s.d.; Ns are centrioles—*yw*=138 (same data as in Fig. 7b), *plk4*^*−*^=188, *WT*=197, *9A*=153, *9DE*=137, pooled from five experiments. (**c**) Model showing how Cep135 is oriented in the centriole, with its N-terminus extending near the centriole wall. Phosphorylation of Cep135 by Plk4 induces a Cep135–Asl interaction that properly positions Asl within the centriole. Comparisons made by Kolmogorov–Smirnov tests. ****P<0.0001, *P≤0.05, n.s., not significant. Scale bar, 1 μm.

These results suggest that the radial positioning of Asl by Cep135 is Plk4-dependent. To test this directly, we examined the position of Asl on centrioles in *plk4* mutants. Although Plk4 is critical for centriole duplication, testes in *plk4* mutant embryos contain a few remnant centrioles in the male germline^49^. The radial position of Asl was measured on these remnants in spermatocytes. Consistent with the Plk4 being important for positioning Asl, the Asl diameter on centrioles is significantly increased in the *plk4* mutant (Fig. 8a,b), sometimes well over 400 nm. Importantly, we found that Asl positioning is partially rescued by expression Cep135^9DE^, but not by Cep135^9A^ in the *plk4* mutant (Fig. 8a,b). This supports a model where Plk4, via Cep135 phosphorylation, properly positions Asl. Therefore, not only is it possible to use the centrosome interactome to identify kinase substrates, but it is also a powerful approach to discover how phosphorylation of centrosome components influences the interaction landscape. This form of phosphor-regulation can then be tested *in vivo*, as we have performed with Cep135 phosphorylation.

## Discussion

As a non-membrane-bound organelle, centrosome assembly must be driven by PPIs. These interactions are likely modified by regulated changes in protein-binding affinity in a cell-type and cell-cycle-dependent manner, which can in turn modulate centrosome behaviour and function. This study focused on identifying PPIs among a core set of conserved centrosome proteins, including proteins of the centriole, the PCM and regulatory kinases. Previous studies suggest that there might be a limited number of PPIs among centrosome proteins used to construct a simple, ultimately static structure. Our data do not support this simplistic view. We have uncovered a large number of direct PPIs, dramatically expanding our understanding of the centrosome interaction landscape. This suggests a much more complex centrosome assembly and regulatory process, which we suggest can be leveraged to perform a variety of specialized tasks dictated by a broad spectrum of cellular requirements.

Although we do not suggest that all the interactions we identify occur *in vivo*, we strongly caution against dismissing interactions because they simply do not support the average location of a protein as determined by SIM using a limited number of cell types. Some, if not most, of the interactions we identified must serve purposes beyond solidifying the final structure of the centrosome, given that we observe interactions between proteins that appear quite distant with light microscopy. For example, we find an HCI between the C-terminus of Cep135, a core centriole protein, and the C-terminus of Cnn, a PCM protein. This interaction is not supported by the average position of these proteins in the final mother centrosome structure by SIM. An alternative model is that these proteins transiently bind during daughter centriole assembly. In fact, a model where PCM plays a role in concentrating centriole proteins to aid centriole assembly has been previously proposed^50^,^51^. Furthermore, the daughter centriole forms adjacent to the proximal end of the mother centriole where the PCM is located. Therefore, the Cep135^c-term^–Cnn^c-term^ interaction might be required for concentrating Cep135 during centriole duplication.

To further illustrate that many interactions might be used transiently, we highlight the localization diversity of Sas-4. Many of the Sas-4 structures we observed differ from the previously reported average position. Furthermore, Sas-4 localization in S2 cells and the developing *Drosophila* wing disc, although similar, are not identical, illustrating a cell-type-specific diversity in its localization. The variety of localizations we observe are consistent with Sas-4's many PPIs with centriole and PCM components, and its roles in centriole duplication and PCM recruitment. Additional support for Sas-4 interacting with different centriole proteins comes from ‘proximity biotin labelling'. The mammalian orthologue of Sas-4, CPAP, was found in close proximity to Asl/Cep152, localized to the outside of the centriole, and Ana2/STIL, localized to the inside of the centriole^29^. Taken together, we propose that interaction diversity begets functional diversity, not only for Sas-4, but for all centrosome proteins.

In addition to centrosomal roles, some interactions may be important at locations away from the centrosome. For instance, some may be important to form cytoplasmic subcomplexes, such as S-CAP^36^ and the PLP–Cnn cytoplasmic ring-like complexes observed in *Drosophila* embryos^43^. These subcomplexes might reorganize their interaction network once delivered to the centrosome, or to other sites, to allow for a different PPI network to assemble. The Golgi, which is known to serve as an MTOC in *Drosophila*^52^,^53^ and mammalian cells^54^, is one example of where unique interactions might occur among centrosome proteins. For example, in the absence of a centriole, PCM proteins might be free to repurpose their PPIs to establish new networks on the Golgi. Finally, a subset of these interactions may be important to construct the centrosome as a non-membrane-bound organelle. The plethora of multivalent interactions we uncovered would provide an ideal mechanism to assist in the segregation of these proteins to a discrete cellular domain, as proposed and supported by *in vitro* reconstitution experiments^18^.

An unexpected finding from our screen was the low frequency with which FL proteins interacted in comparison with protein fragments. This discrepancy might help explain why so few interactions among centrosome proteins were identified in high-throughput Y2H screens. A number of studies have suggested that using protein fragments is advantageous for Y2H^10^,^21^,^55^,^56^. Therefore, the difference in the interactions made by FL protein and protein fragments may be illustrative of centrosome protein regulation and not simply a technical limitation of Y2H. An attractive model is that FL centrosome proteins are unable to interact with the full complement of their partners, perhaps because of an autoinhibited confirmation, which would be relieved by using fragments. Our data set includes many examples that support this model. For instance, Cnn^FL^ and Spd-2^F1^ did not interact in our screen, but Cnn^F1^–Spd-2^F1^ did interact as an HCI. A cellular event, such as Cnn phosphorylation or interaction with another protein(s), could relieve Cnn^FL^ autoinhibition *in vivo* and promote Cnn^F1^–Spd-2^F1^ interaction. In fact, this precisely what has been shown for *C. elegans* Spd-5 (Cnn)-Spd-2 network assembly^18^,^57^.

A second example is the regulated interaction that we identified between Cep135 and Asl. In this case, the N- and C-terminal fragments of Cep135, but not Cep135^FL^, could interact with Asl^C-term^. We go on to show that phosphorylation of Cep135 by Plk4 relieves the autoinhibition present in FL Cep135, allowing it to interact with the Asl^C-term^. In the absence of this interaction in the fly, the positioning of Asl on the centriole is perturbed. Furthermore, Plk4 is critical for this positioning. This is the first example of Plk4, playing a role in constructing or maintaining the organization of centriole proteins outside its well-established role in initiating centriole duplication. While we show that this role is partially via phosphorylation of Cep135, our data suggest that additional unknown Plk4 substrates are important for regulating Asl positioning.

Related to our study on Plk4/Cep135/Asl is a recent report showing that Asl recruitment to newly formed daughter centrioles requires Ana1 as a bridge between Cep135^N-term^ and Asl^C-term^ (ref. 37). Both studies report that the peaks of the fluorescence distributions of Cep135^N-term^ and Asl^C-term^ are separated. However, we note that there is significant overlap between these distributions (Cep135^N-term^ in Fig. 2c, red line; Asl^C-term^ in Fig. 4d) where these proteins could directly interact. Our data indicate that Ana1 might not be required to bridge this distance, since Ana1 is properly positioned on centrioles lacking Cep135, while Asl is not. One model that would synthesize the results from both studies is that Cep135 and Ana1 are required to load Asl on newly formed daughter centrioles. Once recruited, Plk4 could phosphorylate Cep135, allowing it to interact with Asl. This direct Cep135–Asl interaction would then be required to maintain the position of Asl on mature centrioles.

By combining our interactome with *in vivo* experimental evidence in *Drosophila*, we demonstrate how large interaction information can lead to in-depth mechanistic insight into macromolecular assemblies. Integrating protein localization, dynamics and functional data with direct PPI information and mutant analysis, we show that interactions can predict inter- and intramolecular architecture, identify kinase substrates and uncover regulated interactions within the centrosome. Understanding the interaction landscape of the centrosome is a critical foundation needed to gain an understanding of the molecular basis for human diseases caused by dysfunction of centriole, centrosome and cilia proteins. The diversity of centrosome-related diseases, such as microcephally, dwarfism, polycystic kidney disease and many others, can be best explained by loss of specific PPIs, rather than a simple complete loss-of-function stemming from a null mutation. We believe that our study will guide new avenues of centrosome research that focus on the in-depth understanding of the diverse functions of these proteins, and could serve as a framework to explore other complex cellular processes.

## Methods

### Plasmid construction

The sequence encoding the protein fragments used were amplified from cDNA clones by PCR using Phusion (New England Biolabs, Ipswich, MA) and the primers in Supplementary Table 2, and then introduced into Gateway Entry vectors using the pENTr/D-TOPO Kit (Life Technologies, Grand Island, NY). All fragment names and the amino acids of the protein included are in Supplementary Table 2. Gateway reactions were used to recombine cDNAs into the following destination vectors: pUGW (ubiquitin promoter, N-terminal GFP fusion, P-element and mini-white gene sequences for transgenic fly construction; *Drosophila* Genomics Resource Center), pATRW (actin promoter, N-terminal TagRFP; this study), pAWTR (actin promoter, C-terminal TagRFP; this study), pAFHW (actin promoter, N-terminal FLAG and HA fusion; *Drosophila* Genomics Resource Center), pAGW (actin promoter, N-terminal GFP; *Drosophila* Gateway Vector Collection), pAWG (actin promoter, C-terminal GFP; *Drosophila* Gateway Vector Collection), pDEST-pGADT7 (Y2H bait plasmid, GAL4 DNA-binding domain)^58^ and pDEST-pGBKT7-Amp (Y2H prey plasmid, GAL4 Activation domain^59^. Eight of the nine mutations in the *cep135* cDNA were generated by DNA syntheses of the region (GenScript, Piscataway, NJ). The mutant sequence was then incorporated into the cDNA using Gibson cloning^60^. The ninth residue was mutated using the QuickChange II Kit (Agilent Technologies, Santa Clara, CA). Mutations in Ana2 (T242, S318 and S370) were generated using the QuickChange II Kit.

### Centrosome Y2H screen

The screen presented herein is designed following the approach outlined in ref. 20. Since there are multiple advantages to utilize FL proteins, as well as subfragments of proteins in Y2H studies, we utilized both in our screen. We subdivided our proteins using a structure-based approach. We avoided dividing known structural motifs. However, the majority of the proteins in the screen do not have determined structures. In these cases we utilized alignments and structure and motif prediction programmes including Jpred3, Phyre2, the Simple Modular Architecture Research Tool (SMART) and Coils^61^,^62^,^63^,^64^ and avoided making subdivisions in regions of predicted structure or high conservation. In addition to the centriole proteins discussed above, our screen included empty vectors to control against auto-activation, and two coiled-coil regions from non-centrosomal proteins, to control against nonspecific coiled-coil interactions.

Y2H experiments were based on modifications of the Matchmaker Gold system (Clonetech Laboratories, Mountain View, CA) and performed following a significantly modified protocol^20^. All of the constructs listed in Supplementary Table 2 were recombined into both Y2H bait and prey plasmids by Gateway recombination. All bait plasmids were individually transformed into the Y2HGold, a MAT**a** strain, and all prey plasmids into Y187, a MATα strain.

All bait strains were then individually mated to all prey strains in a 96-well format. A volume of 20 μl of an overnight culture of a single bait and a single prey strain were mixed with 100 μl of 2 × Yeast extract Peptone Adenine Dextrose (10 g l^−1^ yeast extract, 20 g l^−1^ peptone, 80 mg l^−1^ adenine and 20 g l^−1^ D-glucose). Plates were incubated for 20–24 h at 30 °C with shaking. Approximately 3 μl of each mating reaction was transferred using a 48-pin multiblot replicator (VP 407AH, V&P Scientific, San Diego, CA) to DDO (SD−Leu−Trp) plates and grown for 5 days to select for diploids carrying bait and prey plasmids. For the initial screen, colonies were then replica-plated to DDO, QDO (SD-Ade-His−Leu−Trp) and DDOXA (DDO +Aureobasidin A, Clonetech Laboratories, +X-α-Gal, Clonetech Laboratories, and Gold Biotechnology, St Louis, MO) plates and grown for 5 days at 30 °C. Colonies were then scored on the basis of growth and development of blue colour on a scale of 0 (no growth/colour) to 3 (robust growth/blue colour). Any pair of protein fragments that revealed an interaction on any test plate was subsequently retested in two to six independent experiments. These retests were performed on DDO, QDO and DDOXA plates, as well as on QDOXA (QDO +Aureobasidin A +X-α-Gal) plates, which are the most stringent plates used. Sixteen proteins or fragments activated the Y2H reporters on their own or failed to function in the system in one direction. One protein, Ana2^FL^, could not be tested in either direction. One protein, Ana1, was not included as full length because of cloning difficulties. None of the centrosome proteins or fragments showed an interaction with the coiled-coil controls under the most stringent conditions. Pairs that interacted at all stringencies, including on QDOXA, are reported interacting in Supplementary Data 1. These interactions are summarized in Supplementary Data 2. Venn diagrams were constructed using Venn Diagram Generator (http://jura/wi/mit.edu/bioc/tools/venn.php).

### Identification of HCIs

To prioritize our studies, we used a simple scoring system to select interactions of high confidence. We assume that polypeptides with fewer interactions are less ‘sticky' and therefore less prone to being false-positives. We calculate the overall likelihood of each protein to interact, which we refer to as the interaction frequency (IF)=number of interactions/total tested interactions. IFs are then used to measure the interaction likelihood of two polypeptides (Poly 1 and Poly 2) by calculating IF^poly1^ × IF^poly2^. Finally, we incorporate the strength of the Y2H interaction by calculating an interaction score (IS)=(IF^poly1^ × IF^poly2^)/Y2H strength (1–3) and display this as a percentage (Supplementary Data 2, IS worksheet). The IS was used to rank all the interactions, with the lowest score indicating the highest confidence. We selected the top 20% (37 interactions) and refer to these as the HCIs (Table 1).

### *Drosophila* cell culture

*Drosophila* S2 cells (Drosophila Genomics Resource Center) were cultured in Sf900II medium (Life Technologies) and split every 3–4 days. Transfections were performed in six-well plates containing a confluent monolayer of cells using Effectene (Qiagen, Venlo, the Netherlands) using 1 μg of each plasmid. All manipulations were performed 48 h post transfection. These cell lines have not been authenticated and were not tested for mycoplasma contamination. However, analysis using both phase and fluorescence microscopy suggests no bacterial contamination.

### Sample preparation and microscopy

Following transfection, S2 cells were plated on #1.5 Concanvalin A-coated coverslips and allowed to attach for 20–30 min. Cells on coverslips were quickly washed twice with PBS and then fixed with 3.7% formaldehyde for 10 min. After fixation, cells were washed three times in PBS and then extracted for 1 min in Karsenti's solution (80 mM Pipes, 5 mM EGTA, 1 mM MgSO_4_ and 0.5% Triton X-100). Cells were then post-fixed in 1 mg ml^−1^ NaBH_4_ for 10 min, and then rehydrated in PBS+0.1% Tween 20. Cells were stained with 4,6-diamidino-2-phenylindole (DAPI; Thermo Fisher Scientific Inc.) for 30 min at room temperature and washed in PBS. Coverslips were then mounted on slides using Vectashield (Vector Laboratories, Burlingame, CA).

For experiments in spermatocytes, testes from wandering third instar larvae were dissected in *Drosophila* S2 media and fixed for 20 min in 9% formaldehyde in PBS. Testes were washed three times in PBS+0.3% Triton X-100 (PBST). Blocking was performed for 1 h at room temperature in 5% normal goat serum with agitation. Guinea pig anti-Asl antibodies (1:1,500; gift from G. Rogers, University of Arizona) were incubated with testes in block overnight at 4 °C. The testes were washed three times for 10 min in PBST. Anti-guinea pig secondary antibodies, conjugated with Alexa 568 (1:500, Life Technologies, Catalogue # A-11075), were incubated with testis for 4–8 h at room temperature, followed by three washes in PBST. Testes were mounted in Vectashield under a #1.5 coverslip. For experiments using wing disks, tissue was dissected from wandering third instar larvae and treated identically to testis tissue.

All imaging analyses are SIM-performed on an OMX4 (GE Healthcare, Issaquah, WA) using immersion oil with a refractive index of 1.516. Images were reconstructed and registered using SoftWoRx 6.1.3 (GE Healthcare).

### Centriole measurements

The position of N-termini was inferred using the following N-terminally tagged GFP/TagRFP proteins in S2 Cells or flies: Sas-4^FL^ (Fig. 2b–d), Cep135^FL^ (Figs 2b–d and 3c and 6f,g), PLP^F3–F5^ (Fig. 4c,d) and Asl^F2–F3^ (Fig. 4c,d). The position of C-termini was inferred using the following C-terminally tagged GFP/TagRFP proteins in S2 Cells or flies: Sas-4^FL^ (2e), Ana2^FL^ (Fig. 2b–d), Cep135^FL^ (Figs 3c and 6g), Asl^F3^ (Fig. 4c,d), PLP^F5^ (Fig. 4c,d) and Ana1^FL^ (Fig. 7c,d). Asl position in Figs 7a,b and 8a,b was determined using an antibody raised against the entire Asl protein.

Image analysis was performed in ImageJ (National Institutes of Health, Bethesda, MD). Linescans were performed across the diameter of centrioles and the resulting data were fit to the sum of two Gaussians (Prism, Graphpad, La Jolla, CA). Ana2 data were fit to a single Gaussian. We report two measurements. The ‘outer edge' measurement, which relies on the full-width at half-maximum, is used to convey the possible maximum outer extension of a protein (Supplementary Fig. 2d). The ‘peak' measurement, which references the two peaks of the Gaussians, conveys the position of the centre of the distribution of a protein (Supplementary Fig. 2d′). The outer edge is also used when comparisons are made with diffraction-limited proteins, as a peak measurement would simply provide the centre of the centriole. Although we calculate the ‘diameter' of the peaks and outer edges, we report the ‘Radius' in Figs 2c,d and 3d and Figs 4d and 6g in keeping with literature standards. For the comparison in Fig. 2c, individual linescans across centrioles were normalized to their peak fluorescence and manually aligned. The average±s.d. at each position was calculated and is presented. The resulting curves were fit as above and the fits are presented.

### Data analysis

All data analyses utilized the Excel (Microsoft, Redmond, WA) and Prism (GraphPad) software. Mean±s.d. is reported for all distributions of data. When comparing normal distributions, we used *t*-tests; Welch's correction was applied when the variances were different. When comparing non-normal distributions, we used the Kolmogorov–Smirnov test. In all experiments, the sample size was not determined *a priori*.

### IP and immunoblotting

For each co-IP, 50 μl of a slurry of Protein-A-conjugated Dynabeads (Life Technologies) was incubated with 0.5 μl rabbit anti-GFP antibodies (clone ab290; Abcam, Cambridge, UK) in PBS with 0.01% Tween 20 for 30 min at 4 °C. *Drosophila* S2 cells, from a single well of a six-well dish, expressing the indicated constructs were harvested using centrifugation, and then lysed in 1 ml RIPA buffer (10 mM Tris-HCl, pH 7.5, 140 mM NaCl, 1 mM EDTA, 0.5 mM EGTA, 1% Triton X-100, 0.1% sodium deoxycholate, 0.1% sodium dodecyl sulfate, 1 mM dithiothreitol (DTT), 1 μg ml^−1^ pepstatin A, 1 μg ml^−1^ leupeptin and 1 mM phenylmethylsulphonyl fluoride (PMSF)) or CLB (50 mM Tris, pH 7.2, 125 mM NaCl, 0.1% Triton X-100, 1 mM DTT, 1 μg ml^−1^ pepstatin A, 1 μg ml^−1^ leupeptin and 1 mM PMSF). Lysates were precleared by centrifugation (5 min at 21,000*g*) at 4 °C. A sample of the cleared lysates was taken and run as ‘input' on immunoblots. The remaining lysate was then added to antibody-bound beads and incubated with mixing for 30 min at 4 °C. Beads were washed two to five times for 5 min each in lysis buffer with mixing and beads were harvested with a magnet in between washes. The beads were transferred to a fresh tube during the final wash. Laemmli buffer sample buffer (65 μl of 2 × ) was added to the beads and the bound material was eluted by boiling for 5 min. IP and input samples were resolved using SDS–PAGE, transferred to polyvinylidene difluoride membranes and detected by western blots using mouse anti-GFP (1:5,000, clone JL-8; Clonetech Laboratories, Catalogue #632381) and mouse anti-FLAG (1:1,000, clone M2; Sigma-Aldrich, St Louis, MO, Catalogue #F1804) primary antibodies, followed by horseradish peroxidase-conjugated anti-mouse secondary antibody (1:5,000, Thermo Fisher Scientific Inc., Catalogue #31430). Detection was performed using SuperSignal West Dura Extended Duration Substrate (Life Technologies) and visualized using a ChemiDoc MP Imaging System (Bio-Rad, Hercules, CA). Some bands in western blots are saturated to allow for detection of lower-intensity bands.

### *In vitro* binding assay

CEP135^F2^ was PCR-amplified from a FL CEP135 cDNA and subcloned into pMAL-c2x (NEB) to generate N-terminal maltose binding protein (MBP)-tagged constructs. The Plk4^F2^ PB1-PB2 domain was PCR-amplified from a Plk4 cDNA and subcloned into pGEX-6p2 (GE Life Sciences). BL21 DE3 *Escherichia coli* were grown at 37 °C to an OD_600_ of 0.6, induced with 0.1 mM isopropyl-b-D-thiogalactoside and cells were shifted to 16 °C for 18 h. Cells were centrifuged for 10 min at 2,100*g*, and then the pellets stored in Buffer A (PBS, 10 mM imidazole, 0.1% β-mercaptoethanol) at −80 °C. Cells were lysed in Buffer A by either sonication or using a cell disruptor (Avestin), centrifuged at 23,000*g* for 20 min at 4 °C and the supernatant was mix with amylose-resin (NEB) or glutathione resin (GE Life Sciences). Resins were washed in Buffer A and eluted with Buffer A+10 mM glutathione or 10 mM maltose. Protein-containing fractions were pooled, dialysed overnight in Buffer B (25 mM HEPES pH 7.4, 150 mM NaCl and 1 mM DTT) for glutathione S-transferase (GST) pull down assays. Purified proteins were also concentrated using Amicon10K Ultra Spin Concentrators (Millipore). GST-PB1-PB2 was immobilized on glutathione, mixed with MBP-CEP135^F2^, rocked at 25 °C for 35 min and pelleted at 500*g* for 1 min. Inputs and pellets were analysed using SDS–PAGE.

### *In vitro* phosphorylation assays

*Drosophila* Plk4 kinase domain+downstream regulatory element (DRE) (amino acids 1–317, ‘Plk4 1–317') C-terminally tagged with FLAG-His_6_ was cloned into the pET28a vector, expressed in BL21 (DE3) bacteria and purified on HisPur resin (Thermo Fisher Scientific Inc.) according to the manufacturer's instructions. GST-tagged constructs of the N-terminal region of *Drosophila* Cep135 (amino acids 1–490 of isoform A, accession NP_648749) were bacterially expressed and purified using glutathione resin (Pierce) according to the manufacturer's instructions. Purified GST, GST-Cep135, Plk4 1–317 and 50 μM total ATP (in some cases, spiked with [γ-^32^P] ATP) were incubated for 1 h at 25 °C in reaction buffer (40 mM Na HEPES, pH 7.3, 150 mM NaCl, 5 mM MgCl_2_, 0.5 mM MnCl_2_, 1 mM DTT and 10% glycerol). Control samples for MS were generated by incubating Cep135 in the absence of Plk4 1–317. Samples were resolved on SDS–PAGE and then Coomassie-stained. Stained gels were then either dried and examined by autoradiography, or selected bands were cut from the gels and processed for tandem MS (MS/MS). Standard procedures were used to process (reduce, alkylate, trypsinize and extract) gel samples before analysis using MS/MS. All MS/MS samples were analysed using Mascot (Matrix Science, London, UK; version 1.4.0.288) to search the database, Mascot5_NCBInr_Drosophila melanogaster, with a fragment ion mass tolerance of 0.80 Da and a parent ion tolerance of 20 PPM. Carbamidomethylation of cysteine was specified as a fixed modification; oxidation of methionine and phosphorylation of serine, threonine and tyrosine were specified as variable modifications. Total coverage was 89% for both Plk4-treated and control Cep135 NT.

### Fly stocks

All experiments were performed in third instar male *Drosophila melanogaster* larvae. The investigator randomly selected 5–30 individuals of the indicated genotype from a stock or cross-containing dozens of genetically identical animals based on visible genetic markers and was therefore not blind to the genotype of individuals. Analysis of *cep135* mutants was conducted using hemizygotes of *cep135*^*C04199*^/*Df(3L)Brd15* (ref. 65). All Cep135 transgenic lines were generated using the Cep135 cDNA, with the indicated mutations, cloned into pUGW, by BestGene Inc. using standard P-element-mediated transformation. The *plk4* mutant was fortuitously generated from a production intermediate for another project. A fragment containing upstream activating sequences from yeast and a fluorescent eye reporter was introduced into the promoter of the *plk4* locus by CRISPR and homologous repair, resulting in a disruption of the promoter of the gene and a >95% loss of *plk4* expression as evidenced by quantitative PCR. The Ana1::tdTomato and Cep135::GFP flies were a gift from Tomer Avidor-Reiss (University of Toledo). Sas-4::GFP flies were a gift from Jordan Raff (University of Cambridge).

### Data availability

The data that support the findings of this study are available from the corresponding author upon request.

## Additional information

**How to cite this article:** Galletta, B. J. *et al*. A centrosome interactome provides insight into organelle assembly and reveals a non-duplication role for Plk4. *Nat. Commun.* 7:12476 doi: 10.1038/ncomms12476 (2016).

## Supplementary Material

Supplementary Information Supplementary Figures 1-5, Supplementary Tables 1-3, Supplementary References

Supplementary Data 1 All Y2H data. A grid of all of the Y2H experiments used to generate the centrosome interactome, including all replicates. Each row includes the result from a single Y2H experiment. Each fragment or full-length protein tested was assigned a number for organizational purposes. This number is used in the columns labeled Y187 strain and Y2HGold strain for prey (AD-fusions) and bait (BD-fusions) respectively. Columns labeled AA start and AA end indicate the amino acids encoded by the DNA included in the Y2H plasmid. All experiments were scored as in Supplementary Figure 1C and the results are in the columns labeled DDO, QDO, DDOXA and QDOXA. The summary result of an entire experiment, as based on growth on all plates, is in the column 'Interacts?'. These interactions are summarized in Supplementary Data 2. The presence of a '1' in the column 'AD autoactivator' or 'BD autoactivator' indicates that the prey or bait construct, respectively, was able to activate the Y2H reporters in the absence of any potential binding partner. Constructs that autoactivated could not be assayed for interaction.

Supplementary Data 2 Highest stringency interaction data A grid of all of the interactions found on the highest stringency plates among all of the tested fragments. The 'Interaction Strength' worksheet shows the growth scored on a 0 - 3 system as in Supplementary Figure 1c. This grid summarizes the findings found from testing a given pair in both directions. If an interaction was found in either direction, the strongest interaction is listed. In cases of marginal interactions (scored 1) the interaction was tested multiple times. If these "1" interactions were identified in = 50% of trials, the score remained a "1". If growth was seen in < 50% of the trials it was scored as a 0. The number of times "1" interactions grew over the number of times tested is reported in parenthesis. The 'Interaction Score' tab reports the measured IS (methods) for interactions of all strengths. The interactions highlighted in green are the HCIs (top 20%).

Supplementary Data 3 All Interaction schematic A graphical representation of all of the interactions identified in the Y2H screen at the highest stringency using the same criteria as in the Supplementary Data 2. Interactions with centriole, PCM and regulator proteins are shown separately for each protein. Interactions scored as 2 or 3 are shown with solid lines. Interactions scored a 1 are shown as dashed lines. Lines terminating at the name of the protein indicate an interaction with the full-length protein. Numbers under schematics are amino acid number. Horizontal lines within schematics indicate the locations where proteins were subdivided

## Figures and Tables

**Table 1:**
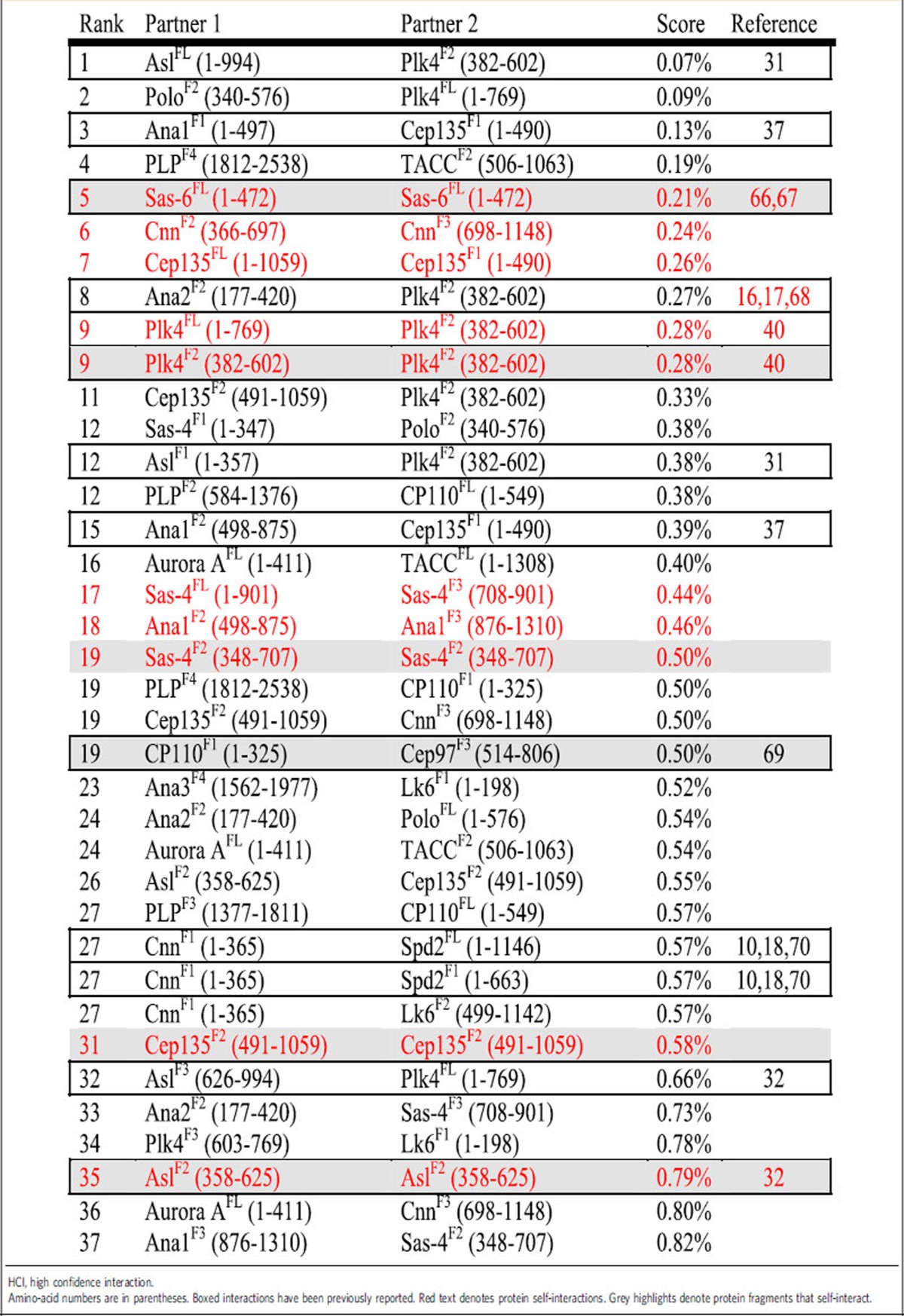
Top 20% (HCIs Methods).
