# Supplementary material for: A centrosome interactome provides insight into organelle assembly and reveals a non-duplication role for Plk4
Source: Nat Commun. 2016 Aug 25;7:12476. doi: 10.1038/ncomms12476 (PMC5007297; doi:10.1038/ncomms12476)
Supplement: Supplementary Information — Supplementary Figures 1-5, Supplementary Tables 1-3, Supplementary References [file ncomms12476-s1.pdf]

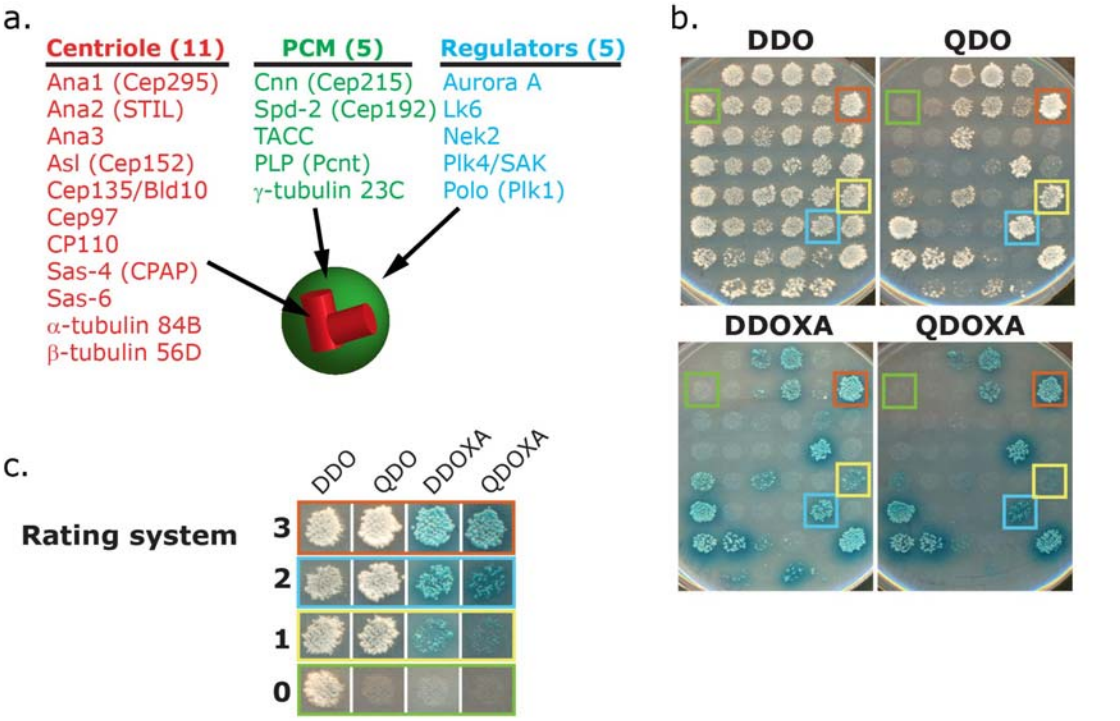

Supplementary Figure 1: Y2H screen design

(**a**) Proteins included in the Y2H screen including centriole proteins, PCM proteins and regulatory kinases. (**b**) Example of a single plate from the Y2H screen. Each colony tests the interaction between a single pair of proteins. DDO plates select for the presence of the Y2H plasmids. QDO plates select for the Y2H plasmids and test for the *ADE2* and *HIS3* reporters, the least stringent interaction condition tested. DDOXA plates select for the Y2H plasmids and test for the MEL1 and *AUR1-C* reporters. QDOXA plates select for the Y2H plasmids and tests for all four reporters, the most stringent conditions for interaction tested. Colored boxes are examples of colonies to highlight our rating system in c. (**c**) Example of the rating system used to score colonies. Column 1 – DDO plate, Column 2 – QDO plate, Column 3 - DDOXA plate, Column 4 – QDOXA plate. The numbers indicate the relative amount of growth and blue color observed on the QDOXA plate (column 4).

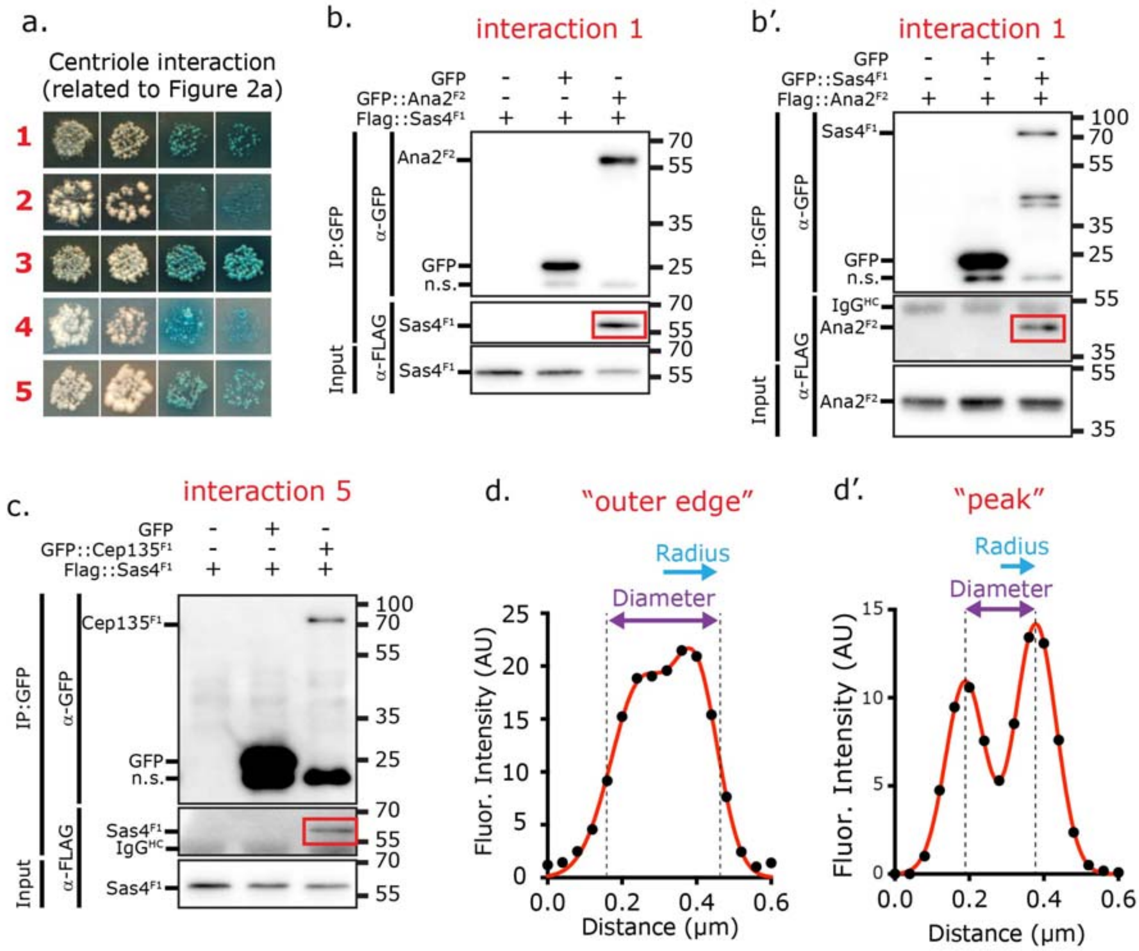

Supplementary Figure 2: Centriole interaction and distance measurement details

(**a**) Results of Y2H interactions among the core centriole proteins. Red numbers refer to interactions indicated in Figure 2a. Y2H data is arranged as in S1c. (**b and b'**). Confirmation of Sas-4^{F1} (aa 1 - 347) and Ana2^{F2} (aa 176 - 420) (interaction #1 from Figure 2a and S2a) by co-IP in both directions. Red box highlights the co-IPed protein. (**c**) Confirmation of Sas-4^{F1} and Cep135^{F1} (aa 1 - 490) interaction #5 from Figure 2a and S2a) by co-IP. Red box highlights the co-IPed protein. (**d and d'**). Examples of line-scans across centrioles. Black circles indicate data points. Red line is the fit to the sum of two Gaussians. Diameters (purple arrows) and Radii (blue arrows) are indicated for the Outer Edge (d) and Peak (d') measurements.

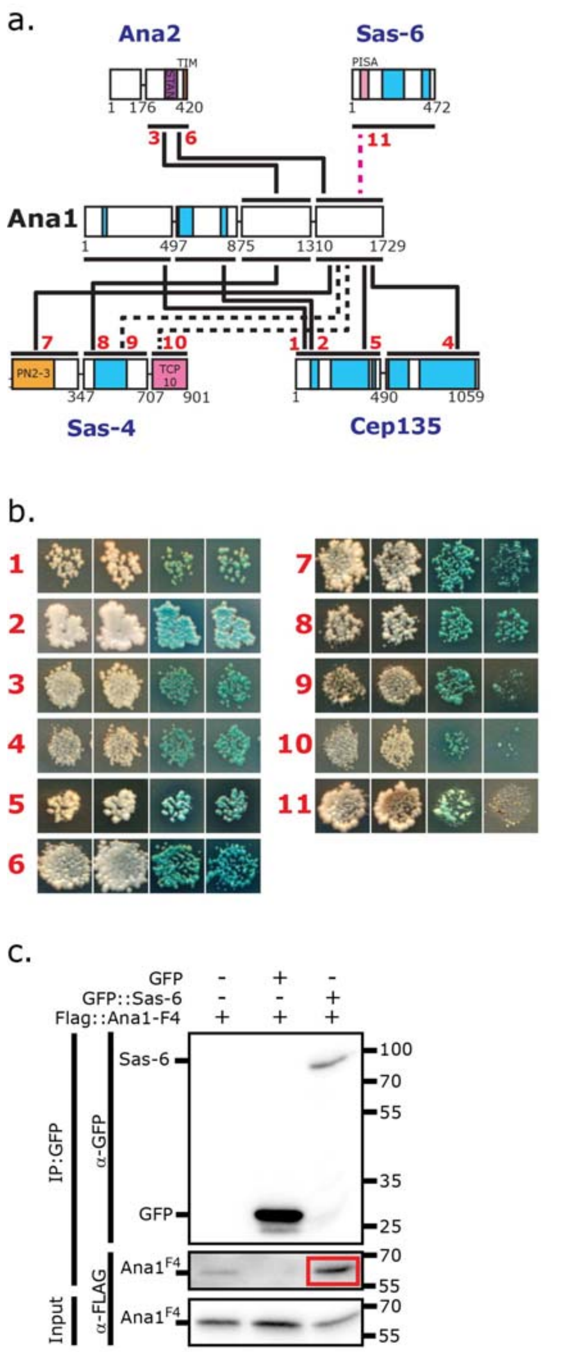

Supplementary Figure 3: Ana1 interacts with several core centriole proteins.

(a) All 11 interactions identified between Ana1 and the core centriole proteins. Schematic as in Fig. 2a. Red numbers refer to interactions tested in b. (**b**) Y2H data for all interactions between Ana1 and core centriole proteins. Red numbers refer to interactions indicated in (a). Y2H data is arranged as in S1c. (**c**) Ana1-Sas6 interaction (#11) shown by co-IP. Red box highlights co-IPed protein. Sizes are in kD.

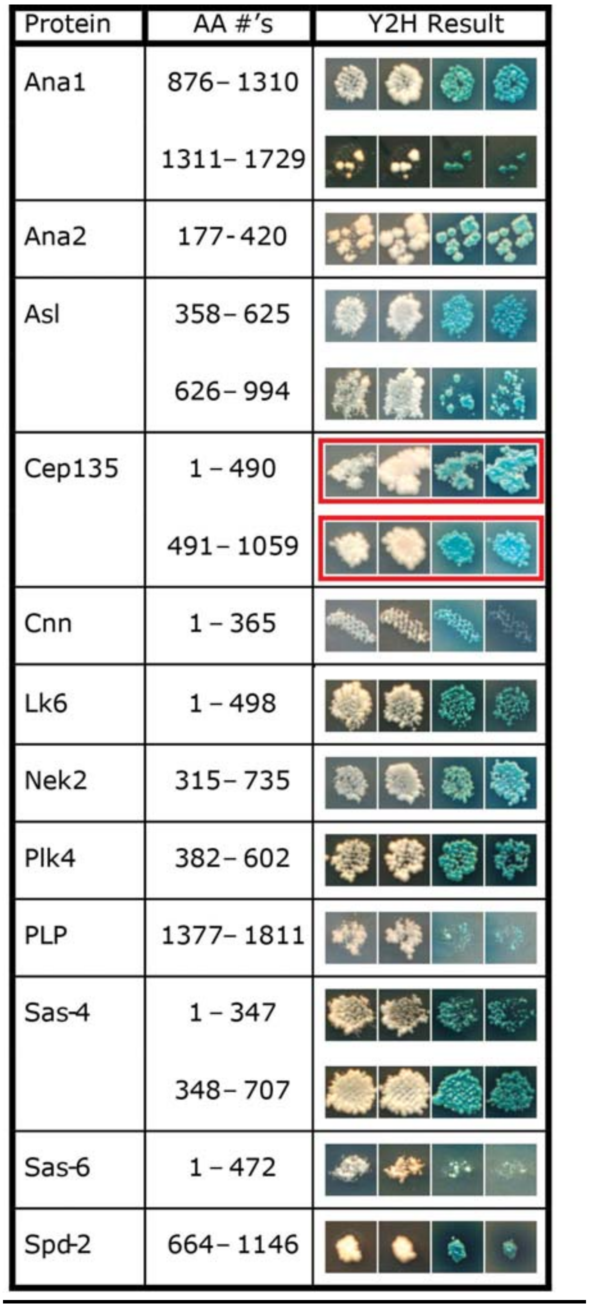

<u>Supplementary Figure 4</u>: Y2H data for protein multimerization (self-association) domains

Related to Fig. 3a. Y2H data for all of the self-associations seen among fragments in the screen. The amino acid numbers of the fragment are listed (middle column). Y2H data (right column) is arranged as in S1c. Red boxes highlight the self-associations made by Cep135.

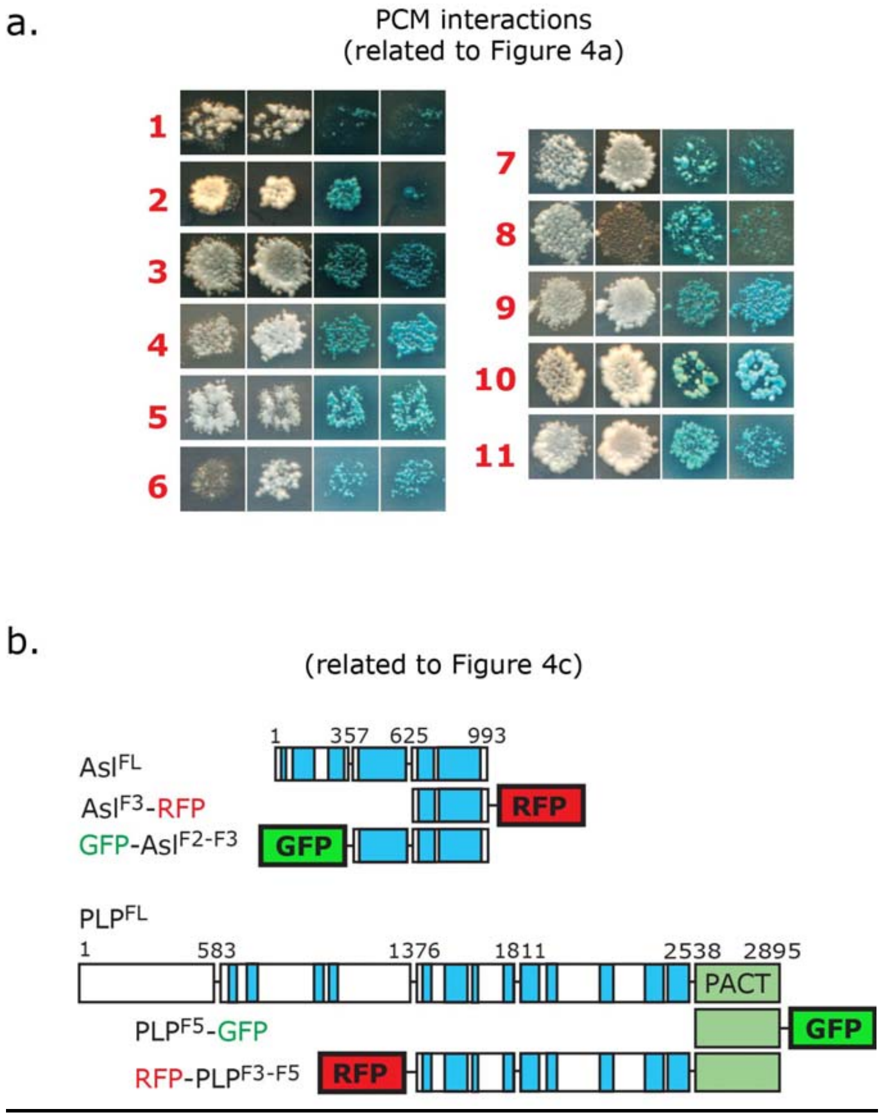

<u>Supplementary Figure 5</u>: PCM interaction and details of bridging protein constructs

(**a**) Y2H data for all of the interactions seen among PCM proteins in the screen. Red numbers refer to Fig. 4a. (**b**) Schematics of Asl and PLP constructs used in Figure 4c, d. Blue regions are predicted coiled-coils. Horizontal black lines indicated the locations where the proteins were subdivided for the screen. Numbers are amino acids.

Supplementary Table 1. Centrosome proteins used in Y2H screen. The *H. sapiens* and *C. elegans* orthologs are listed. n.f. = not found

D. melanogaster	*H. sapiens*	*C. elegans.*
Ana1	Cep295	n.f.
Ana2	STIL	SAS-5
Ana3	Rotatin	n.f.
Asterless	Cep152	n.f.
Aurora A	Aurora A	AIR-1
Centrosomin (Cnn)	CDK5RAP2/Cep215	SPD-5
Cep135/Bld10	Cep135	n.f.
Cep97	Cep97	n.f.
CP110	CP110	n.f.
Lk6	MKNK1,2	MNK-1
Nek2	Nek2	n.f.
Plk4/SAK	Plk4	ZYG-1
PLP	Pericentrin, Kendrin	n.f.
Polo	Plk1	PLK-1/2
Sas-4	CPAP	SAS-4
Sas-6	hSas6	SAS-6
Spd2	Cep192	SPD-2
TACC	TACC	TAC-1
**α**-tubulin 84B	**α**-tubulin	TBA
**β**-tubulin 56D	**β**-tubulin	TBB
**γ**-tubulin 23C	**γ**-tubulin	TBG-1

Supplementary Table 2. Protein fragments used in Y2H screen. The first and last amino acid (AA) included in each protein fragment is indicated. The primers used to amplify the regions encoding these fragments from cDNA clones are indicated.

Fragment Name	First AA	Last AA	Forward Primer (5′ – 3′)	Reverse Primer (5′ – 3′)
Ana1^{F1}	1	497	caccatggctctgcagctaacagtaaatggaaag	cggtggagcagtaatagcgggaccgag
Ana1^{F2}	498	875	caccatgagcactgctacgcaatccactgtgtcc	tggtgtgctgtcagttgagcccagggg
Ana1^{F3}	876	1310	caccatgagagacgatgagccaaagccgacggaac	cgtttccacttctgtaatggtcgacag
Ana1^{F4}	1311	1729	caccatgccaggacaatcacatattgtggccgcc	ttttcggggcgactttcgatgcgttgtc
Ana2^{F1}	1	176	caccatgtttgttcccgaaacggaggatatgc	ttttcgcggaggagatgggttgaaggg
Ana2^{F2}	177	420	caccatgtcactgccccaggttgtctattccgac	caacagcttcggctggttcctgatattctc
Ana2^{FL}	1	420	caccatgtttgttcccgaaacggaggatatgc	caacagcttcggctggttcctgatattctc
Ana3^{F1}	1	713	caccatgagtaccaaacagagcccggccctc	tggtggttgaattatgcagagatcgttggc
Ana3^{F2}	714	1041	caccatgttctgttatagaagcattttcatcaaatgctc	gcgaatgcatgttacgcacagatccag
Ana3^{F3}	1042	1560	caccatgttcagccagccacatgctatcaacggac	gatcgcatggtttggcctttgcttattg
Ana3^{F4}	1562	1977	caccatgcaggccactaatgcggctattataattttcc	ttgtcgcatcgactccatcagtttctcc
Ana3^{FL}	1	1977	caccatgagtaccaaacagagcccggccctc	ttgtcgcatcgactccatcagtttctcc
Asl^{F1}	1	357	caccatgaacacgccaggtataagcctctttc	aggcgattcgtccgcctgatgctgttg
Asl^{F2}	358	625	caccatgactagtcggctaagtcaaatgggtgg	ttcgcgcttttcccgctcgctcctttg
Asl^{F3}	626	994	caccatggaagcagttgcggtggccaaagaaag	gctgtgaccattgcctttgggctttc
Asl^{FL}	1	994	caccatgaacacgccaggtataagcctctttc	gctgtgaccattgcctttgggctttc
Aurora A^{FL}	1	411	caccatgtcccatccgtctgaccatgtgctg	ctgcgtgtgcgccaggatccaaggatg
Cep135^{F1}	1	490	caccatgaatatcaacgatggtgactttaaagacc	ggggggcggcagtgtctcatactgcac
Cep135^{F2}	491	1059	caccatgacagcttcatccattacctccaccgttac	aagagtcttcgatggcacccgggggctaac
Cep135^{FL}	1	1059	caccatgaatatcaacgatggtgactttaaagacc	aagagtcttcgatggcacccgggggctaac
Cep97^{F1}	1	351	caccatgagtggcgacgaaagtggcgaggag	gctgctgttcccgtgatagctattcac
Cep97^{F2}	352	513	caccatgaacaatagcattgttagcgacaatggatc	cggtgcctgcatgttgtccgcgctgc
Cep97^{F3}	514	806	caccatggagaaactccagcaatcggctgttttgg	tggatctttatcaagattttccgagtc
Cep97^{FL}	1	806	caccatgagtggcgacgaaagtggcgaggag	tggatctttatcaagattttccgagtc
CG42673^{CC}	580	626	caccaatcttggcagcatcaatcagc	tgccgcctgcgtttgttg
Cnn^{F1}	1	365	caccatggaccagtctaaacaggttttgcgg	tggcgaaattaccgtcttagcgcagtc
Cnn^{F2}	366	697	caccatgtcctccagcggccgttccatgagtgac	aggcgctgccaaactgttggaggtttc
Cnn^{F3}	698	1148	caccatggagcaggcgatcagcgagtcggagagc	taactcattctccatgtttgagcgaac
Cnn^{FL}	1	1148	caccatggaccagtctaaacaggttttgcgg	taactcattctccatgtttgagcgaac
CP110^{F1}	1	325	caccatggatgcgacgtgggcaatggagcag	gccatgctcgtccagcgccc
CP110^{F2}	326	549	caccagtgctttggaagccagccgcaaggc	gggcttcttgcgattgcccagccgcttgg
CP110^{FL}	1	549	caccatggatgcgacgtgggcaatggagcag	gggcttcttgcgattgcccagccgcttgg
Lk6^{F1}	1	498	caccatggtggagcccaagtccgggacagc	cgagcgattgcgagtgtaatgacccgg
Lk6^{F2}	499	1142	caccatgcagcgcaatcccgcttcctccttgtgc	accactggcggtggccggctcgcag
Lk6^{FL}	1	1142	caccatggtggagcccaagtccgggacagc	accactggcggtggccggctcgcag
Nek2^{F1}	1	314	caccatgagcggagaggaatctgccggcatg	gtcctcgaaaagcctggcacccgaagg
Nek2^{F2}	315	735	caccatggaagaggaagatggagttcatccagag	gatcaccatctggtcgcgcgttgttag
Nek2^{FL}	1	735	caccatgagcggagaggaatctgccggcatg	gatcaccatctggtcgcgcgttgttag
Plk4/SAK^{F1}	1	381	caccatgttatccaatcgggcgtttggagaaac	ttccttaagagatgcctgtttggaattgg
Plk4/SAK^{F2}	382	602	caccatggatcgcatttcggtgccaccattgaatac	gggttgtacatctgttataggtcttcg
Plk4/SAK^{F3}	603	769	caccatggctcagagattggatggcctacgagatac	aagaagcatgcgattataataaggcgttg
Plk4/SAK^{FL}	1	769	caccatgttatccaatcgggcgtttggagaaac	aagaagcatgcgattataataaggcgttg
PLP^{F1}	1	583	caccatggccattaatattgctttatttacg	aggcggatcctgctcctcttcc
PLP^{F2}	584	1376	caccatgtccctctccttggatgagtc	tggaggtagggaggaatgtgtttttccc
PLP^{F3}	1377	1811	caccatggatcttcaagagcatgcggg	atcccgctcatgttgaagctccacg
PLP^{F4}	1812	2538	caccatgacgctgcagggtcgtatggagg	ttcattgaagtgttccaactctgtttcgg
PLP^{F5}	2539	2895	caccatgcgtttaaccctgcaagccag	atgatgccgcgcatgcgctc
PLP^{FL}	1	2895	caccatggccattaatattgctttatttacg	atgatgccgcgcatgcgctc
Polo^{F1}	1	339	caccatggccgcgaagcccgaggataagagc	ggtaatggcgtcgtgcagattggcc
Polo^{F2}	340	576	caccatggcgtcagcgcaggtgtgccgccacagc	tgtgaacatcttctccagcattttcctaag
Polo^{FL}	1	576	caccatggccgcgaagcccgaggataagagc	tgtgaacatcttctccagcattttcctaag
Sas-4^{F1}	1	347	caccatgcaggaggctggcgaaagtcctgttg	ctcctgcagttcctgcaccagcatctg
Sas-4^{F2}	348	707	caccatggggacgaggaggacaccgaaccg	agccggtggggggtagcagctttcgg
Sas-4^{F3}	708	901	caccatggccaactccagcagtgactttaagcgg	atacttggcatagtctgtgtccattatgag

Fragment Name	First AA	Last AA	Forward Primer (5′ – 3′)	Reverse Primer (5′ – 3′)
Sas-4^{FL}	1	901	caccatgcaggaggctggcgaaagtcctgttg	atacttggcatagtctgtgtccattatgag
Sas-6^{FL}	1	472	caccatgtggcctccagggagcgaggatagc	tcgccgattttctttgcccgtgggtattg
Spd2^{F1}	1	663	caccatggacagtagcagtggaagccaagg	tgtgaatccgctggtggaactggcctc
Spd2^{F2}	664	1146	caccatggcgagtggaagacgtgggttgggaacc	aaatttaaaactaatcgggacactgatgcg
Spd2^{FL}	1	1146	caccatggacagtagcagtggaagccaagg	aaatttaaaactaatcgggacactgatgcg
Syntaxin 1A^{CC}	33	117	caccgacgacttcttcgcccag	aattctcagatccgccgacg
TACC^{F1}	1	505	caccatggatttcctcgcgaatatcatcaaaag	gcttgaaacgggagaatgagcattaatttgc
TACC^{F2}	506	1063	caccatgaaatttttcttaaaactctgcgttcaaaatgatacgg	tttaacatgctttttttgttctatccacagg
TACC^{F3}	1064	1308	caccatgagtgtggacgtcattgataacgattgc	actactaccaccttgtcccttgccatag
TACC^{FL}	1	1308	caccatggatttcctcgcgaatatcatcaaaag	actactaccaccttgtcccttgccatag
αTub84B^{FL}	1	450	caccatgcgtgaatgtatctctatcc	gtactcctcagcgccctcaccctcgcc
βTub56D^{FL}	1	456	caccatgagggaaatcgttcacatcc	Gttctcgtcgacctcagcctcctgc
γTub23C^{FL}	1	475	caccatgccaagtgaaataattactttgcagcttg	ggaaccggcgctggtcacagatcgac

<u>Supplementary Table 3a.</u> Direct PPIs identified in large scale Y2H screens in *Drosophila* among the centrosome proteins included in this study. Highlighted interactions were also found in this study.

Partner 1	Partner 2	Reference
Cnn	**β**-tubulin 56D	1
Lk6	**β**-tubulin 56D	1

<u>Supplementary Table 3b.</u> Direct PPIs identified in large scale Y2H screens in *C. elegans* among the centrosome proteins included in this study. Highlighted interactions were also found in this study. *Drosophila* protein names are in parentheses.

Partner 1	Partner 2	Reference
SAS-4	SAS-4	2
SAS-5 (Ana2)	SAS-5 (Ana2)	2
SAS-5 (Ana2)	SAS-6	2
SPD-2	SPD-5 (Cnn)	2
SPD-5 (Cnn)	SPD-5 (Cnn)	2
AIR-1 (Aurora A)	SPD-5 (Cnn)	2
PLK-1 (Polo)	SAS-4	2
PLK-1 (Polo)	SPD-2	2
PLK-1 (Polo)	TBB-1 (**β**-tubulin 56D)	2
ZYG-1 (Plk4)	TAC-1 (TACC)	2

Supplementary Table 3c. Direct PPIs identified in large scale Y2H screens in *Homo sapiens* among the centrosome proteins included in this study. Highlighted interactions were also found in this study.

Partner 1	Partner 2	Reference
Plk4	Plk4	3,4

<u>Supplementary Table 3d.</u> Proteins that co-purrified in a large scale pull down / mass spec screen in *Drosophila* among the centrosome proteins included in this study. Highlighted interactions were also found in this study. Note: The interactions identified in this screen may be indirect.

Partner 1	Partner 2	Reference
Sas-4	Sas-6	5

<u>Supplementary Table 3e.</u> Proteins that co-purrified in a large scale pull down / mass spec screen in *Homo sapiens* among the centrosome proteins included in this study. *Drosophila* protein names are in parentheses.
Highlighted interactions were also found in this study. Note: The interactions identified in this screen may be indirect.

Partner 1	Partner 2	Reference
Cep135	Cep135	6
Cep152 (Asl)	Cep152 (Asl)	6
Cep295 (Ana1)	CPAP (Sas-4)	6
CPAP (Sas-4)	CP110	6
CPAP (Sas-4)	CPAP (Sas-4)	6
Sas-6	Sas-6	6
CDK5RAP2 (Cnn)	CDK5RAP2 (Cnn)	6
CDK5RAP2 (Cnn)	Pericentrin (PLP)	6
Plk4	Cep152 (Asl)	6
Plk1 (Polo)	Plk1 (Polo)	6

Supplementary Table 3f. Direct PPIs identified in small-scale studies in *Drosophila* among the centrosome proteins included in this screen. Only interactions identified by Y2H or *in vitro* using recombinant proteins are included. Highlighted interactions were also identified in this study.

Partner 1	Partner 2	Reference
Ana1	Cep135	7
Ana1	Asl	7
Ana2	Ana2	8
Ana2	Plk4	9
Ana2	Sas-4	10
Ana2	Sas-6	11
Asl	Asl	12
Asl	Plk4	12,13
Asl	PLP	14
Asl	Spd-2	15
Sas-6	Sas-6	8
Cnn	γ-Tubulin 23C	16
Cnn	PLP	14,17
Cnn	Spd-2	15
PLP	PLP	14
PLP	Spd-2	14
Aurora A	Cnn	16
Aurora A	TACC	18
Plk4	Plk4	19,20

Supplementary References

1. Giot, L. et al. A protein interaction map of Drosophila melanogaster. *Science* **302**, 1727-1736 (2003).
2. Boxem, M. et al. A protein domain-based interactome network for C. elegans early embryogenesis. *Cell* **134**, 534-545 (2008).
3. Rolland, T. et al. A proteome-scale map of the human interactome network. *Cell* **159**, 1212-1226 (2014).
4. Rual, J. F. et al. Towards a proteome-scale map of the human protein-protein interaction network. *Nature* **437**, 1173-1178 (2005).
5. Guruharsha, K. G. et al. A protein complex network of Drosophila melanogaster. *Cell* **147**, 690-703 (2011).
6. Hein, M. Y. et al. A human interactome in three quantitative dimensions organized by stoichiometries and abundances. *Cell* **163**, 712-723 (2015).
7. Fu, J. et al. Conserved molecular interactions in centriole-to-centrosome conversion. *Nat Cell Biol* **18**, 87-99 (2016).
8. Cottee, M. A. et al. The homo-oligomerisation of both Sas-6 and Ana2 is required for efficient centriole assembly in flies. *Elife* **4**, e07236 (2015).
9. Dzhindzhev, N. S. et al. Plk4 Phosphorylates Ana2 to Trigger Sas6 Recruitment and Procentriole Formation. *Curr Biol* **24**, 2526-2532 (2014).
10. Zheng, X. et al. Conserved TCP domain of Sas-4/CPAP is essential for pericentriolar material tethering during centrosome biogenesis. *Proc. Natl. Acad. Sci. U.S.A.* **111**, E354-63 (2014).
11. Stevens, N. R., Dobbelaere, J., Brunk, K., Franz, A. & Raff, J. W. Drosophila Ana2 is a conserved centriole duplication factor. *J Cell Biol* **188**, 313-323 (2010).
12. Klebba, J. E. et al. Two Polo-like kinase 4 binding domains in Asterless perform distinct roles in regulating kinase stability. *J Cell Biol* **208**, 401-414 (2015).
13. Dzhindzhev, N. S. et al. Asterless is a scaffold for the onset of centriole assembly. *Nature* **467**, 714-718 (2010).
14. Richens, J. H. et al. The Drosophila Pericentrin-like-protein (PLP) cooperates with Cnn to maintain the integrity of the outer PCM. *Biol Open* **4**, 1052-1061 (2015).
15. Conduit, P. T. et al. A molecular mechanism of mitotic centrosome assembly in Drosophila. *Elife* **3**, e03399 (2014).
16. Terada, Y., Uetake, Y. & Kuriyama, R. Interaction of Aurora-A and centrosomin at the microtubule-nucleating site in Drosophila and mammalian cells. *J Cell Biol* **162**, 757-763 (2003).
17. Lerit, D. A. et al. Interphase centrosome organization by the PLP-Cnn scaffold is required for centrosome function. *J Cell Biol* **210**, 79-97 (2015).
18. Giet, R. et al. Drosophila Aurora A kinase is required to localize D-TACC to centrosomes and to regulate astral microtubules. *J Cell Biol* **156**, 437-451 (2002).
19. Cunha-Ferreira, I. et al. Regulation of autophosphorylation controls PLK4 self-destruction and centriole number. *Curr Biol* **23**, 2245-2254 (2013).
20. Klebba, J. E. et al. Polo-like kinase 4 autodestructs by generating its Slimb-binding phosphodegron. *Curr Biol* **23**, 2255-2261 (2013).
