# Supplementary material for: A centrosome interactome provides insight into organelle assembly and reveals a non-duplication role for Plk4
Source: Nat Commun. 2016 Aug 25;7:12476. doi: 10.1038/ncomms12476 (PMC5007297; doi:10.1038/ncomms12476)

Ana1 Interactions

Centriole

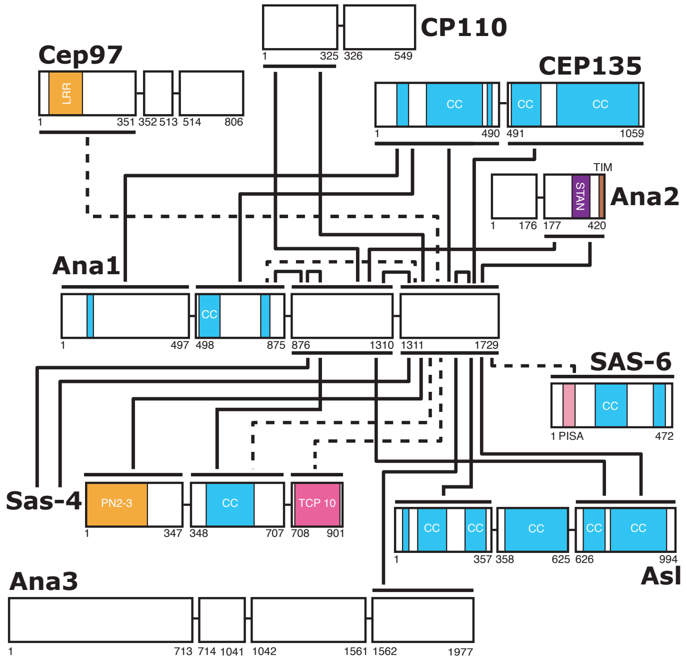

PCM

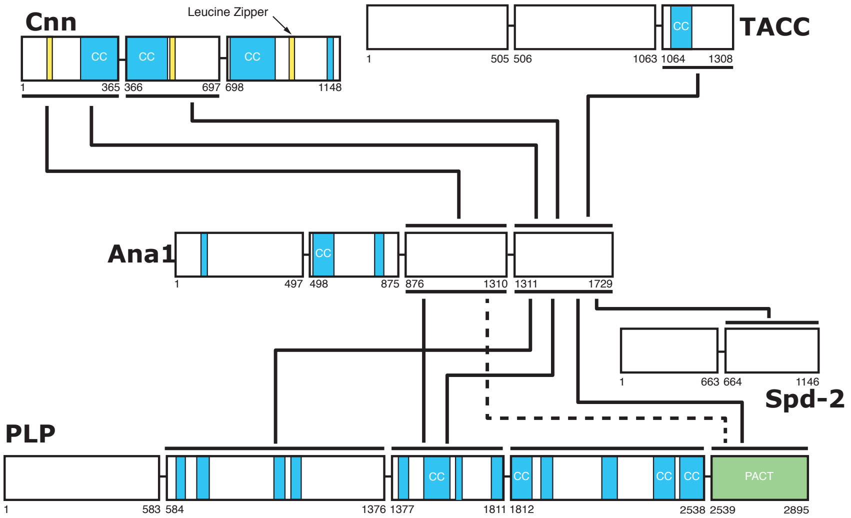

Regulators

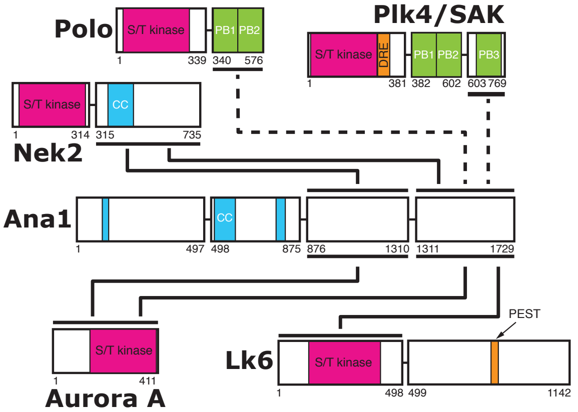

Ana2 Interactions

Centriole

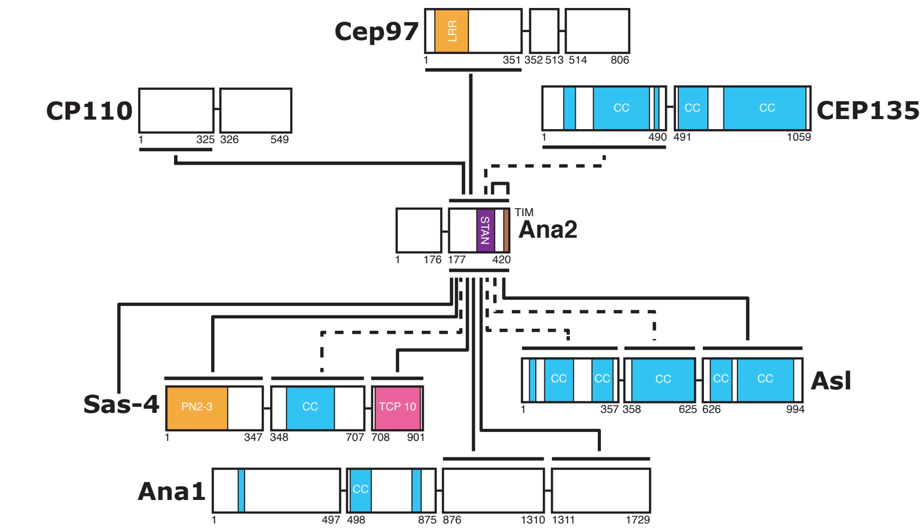

PCM

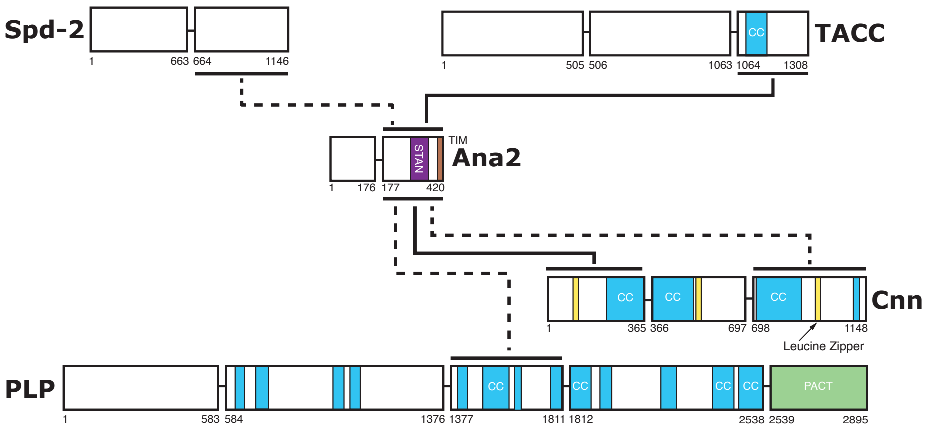

Regulators

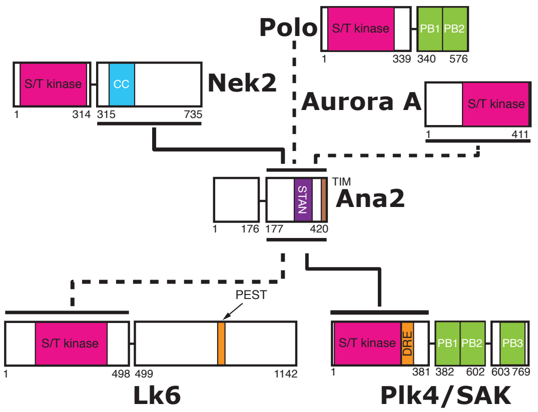

Ana3 Interactions

Centriole

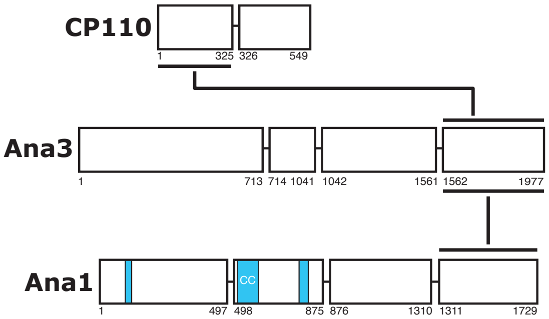

PCM

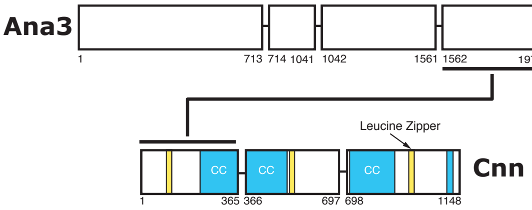

Regulators

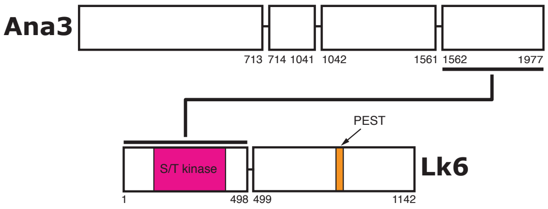

Sas-4 Interactions

Centriole

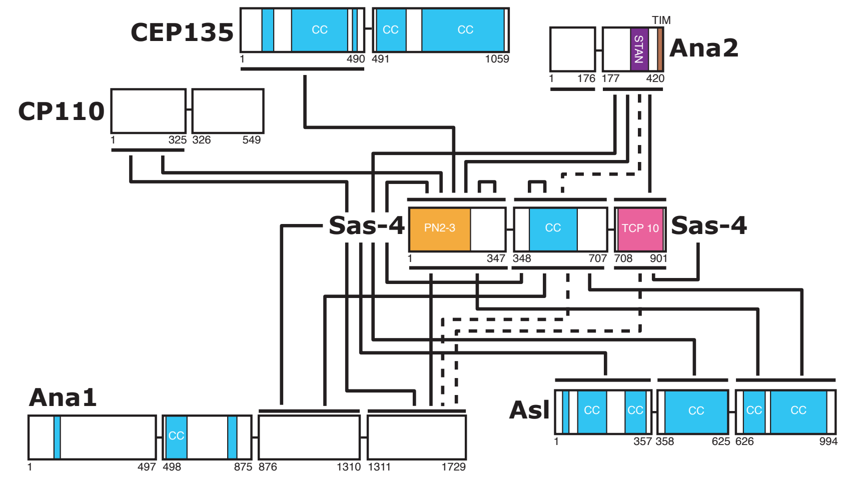

PCM

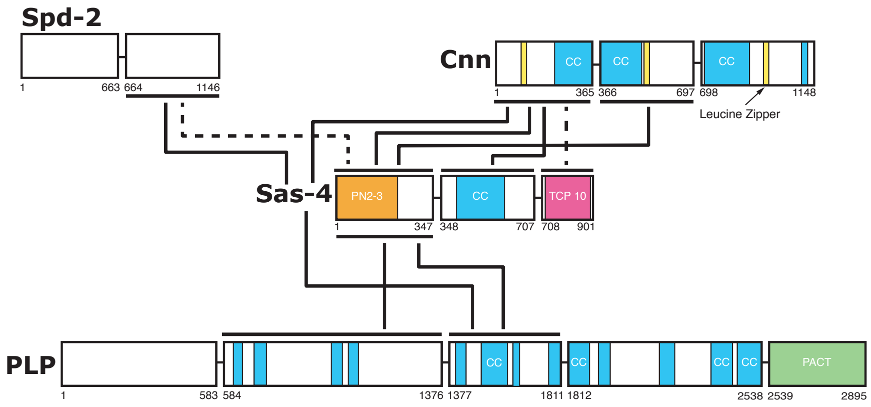

Regulators

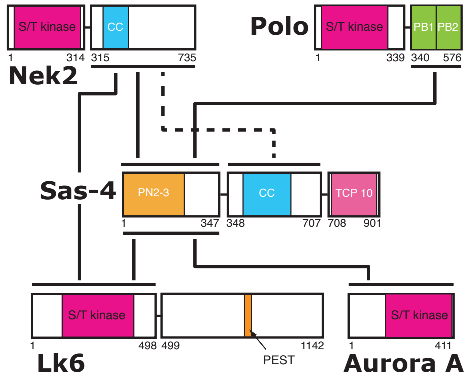

Sas-6 Interactions

Centriole

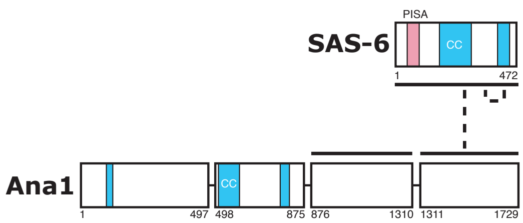

PCM

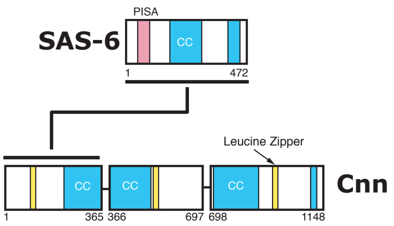

Regulators

Asl Interactions

Centriole

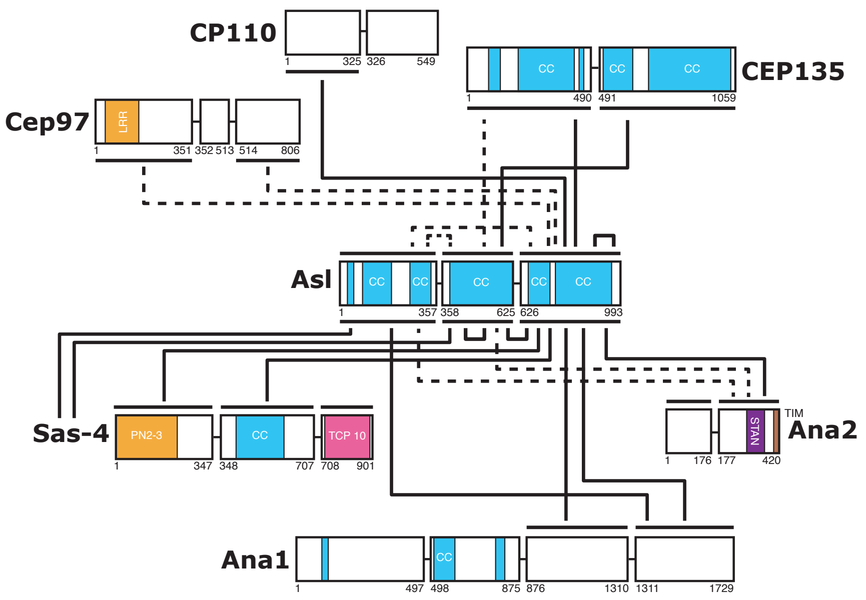

PCM

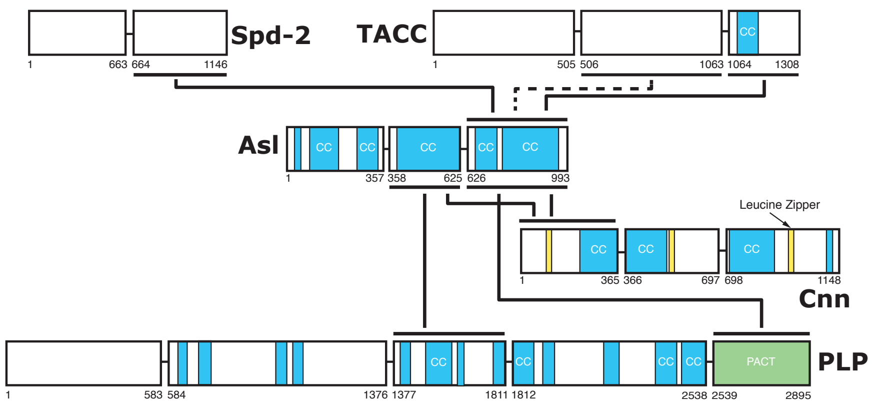

Regulators

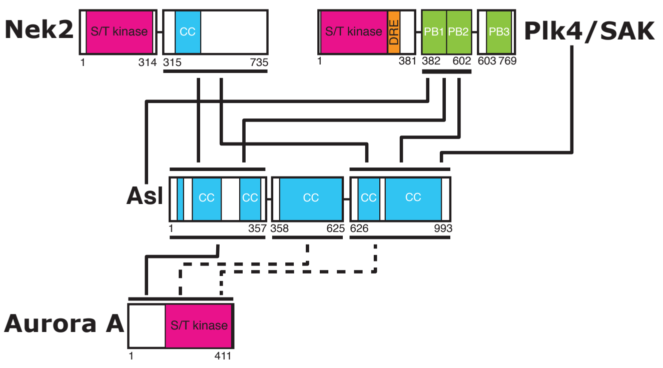

PLP Interactions

Centriole

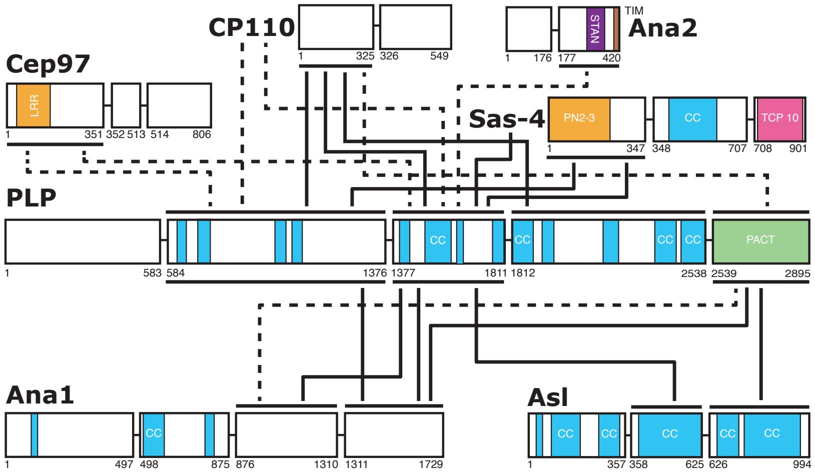

PCM

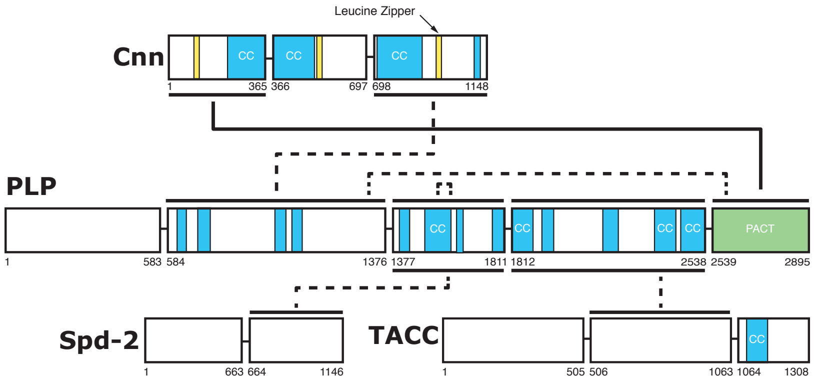

Regulators

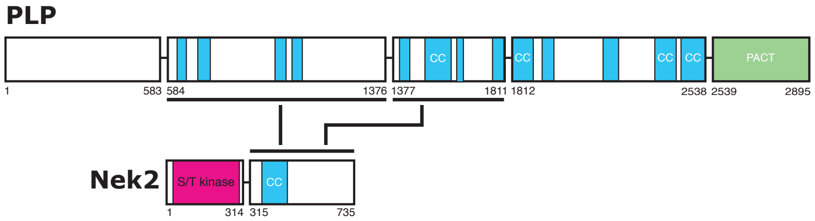

Cep135 Interactions

Centriole

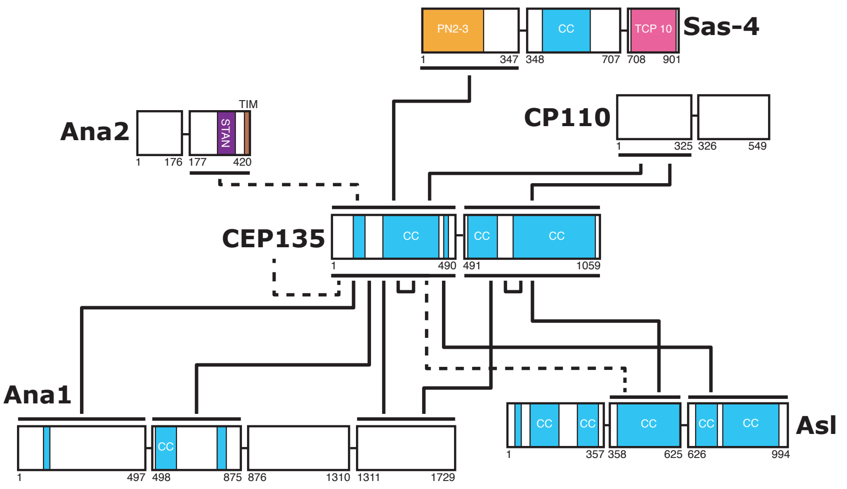

PCM

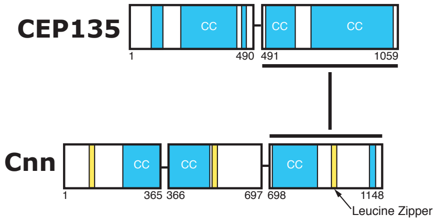

Regulators

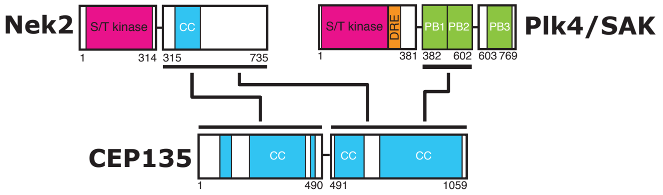

CP110 Interactions

Centriole

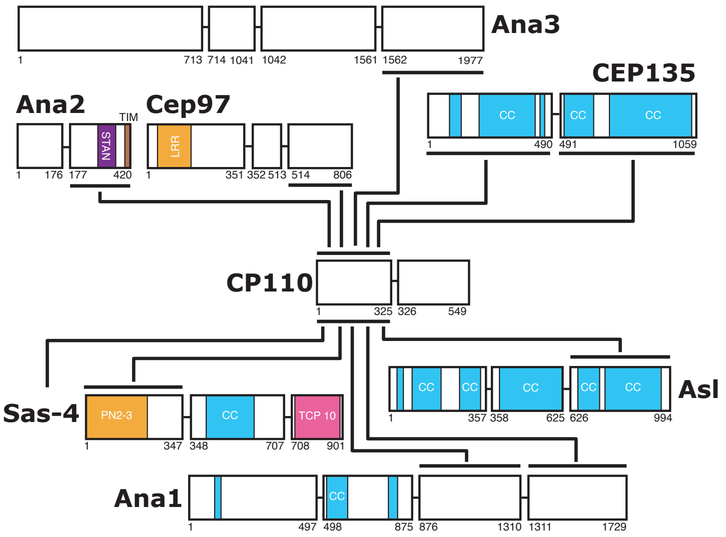

PCM

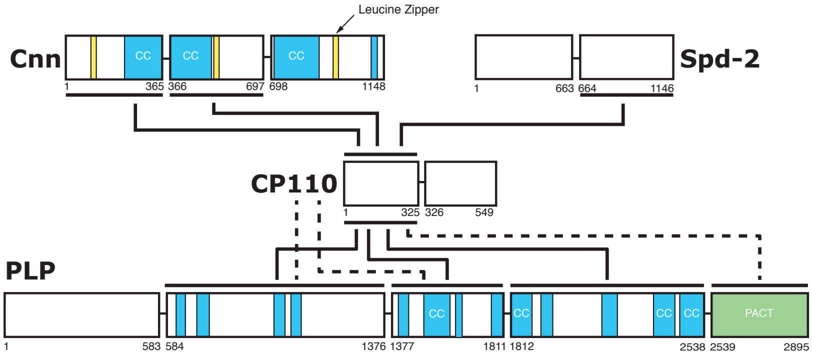

Regulators

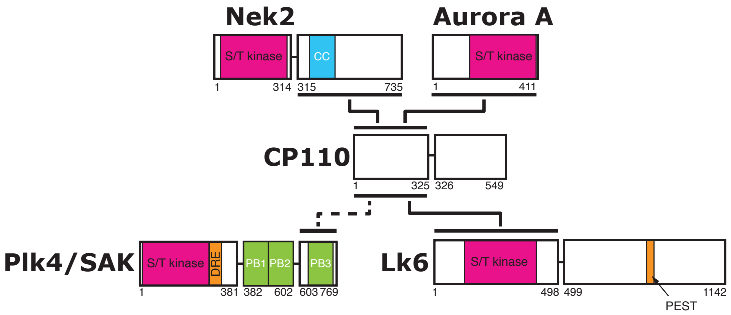

Cep97 Interactions

Centriole

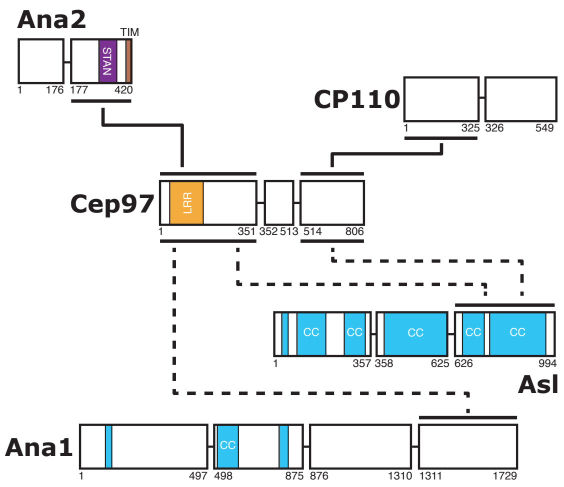

PCM

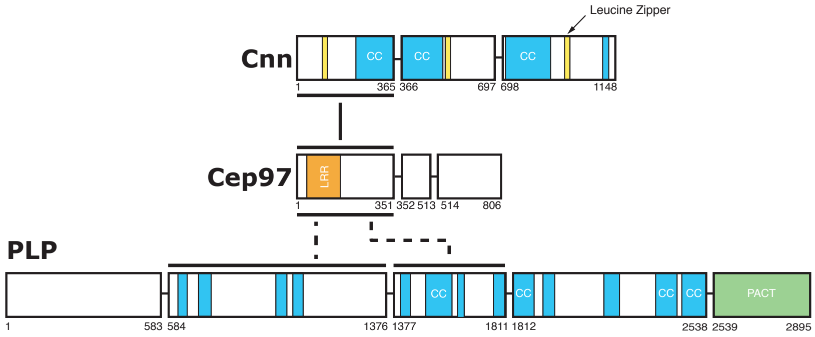

Regulators

Polo Interactions

Centriole

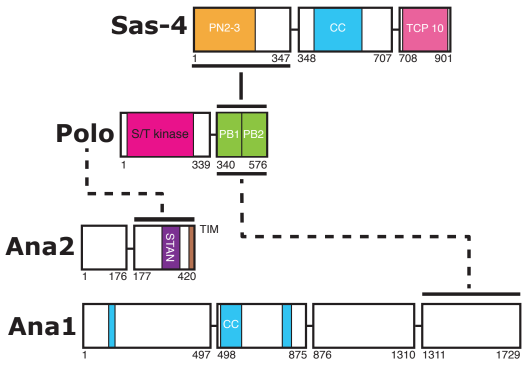

PCM

Regulators

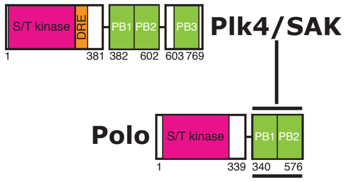

Plk4/SAK Interactions

Centriole

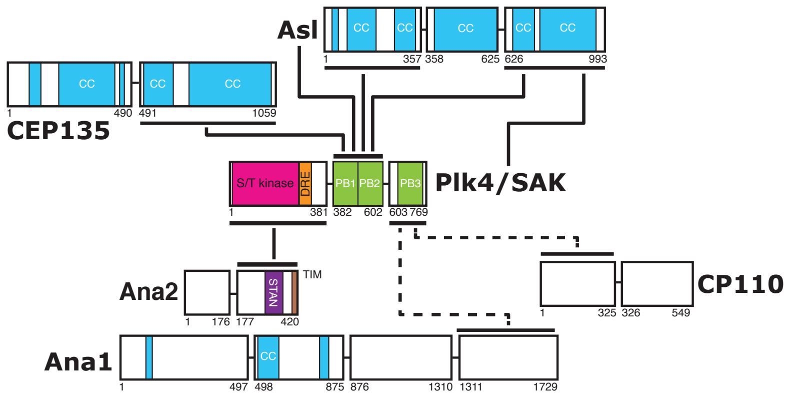

PCM

Regulators

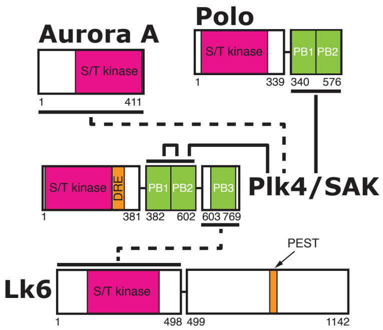

Aurora A Interactions

Centriole

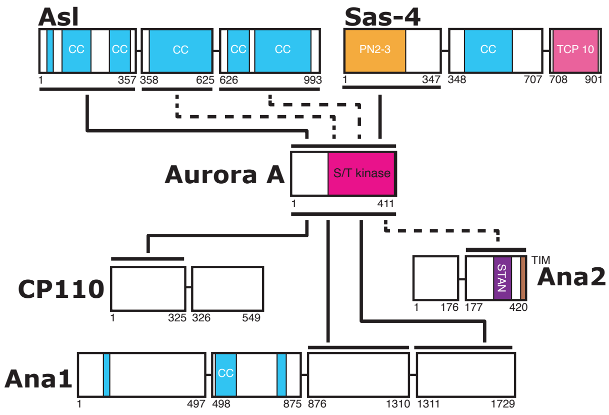

PCM

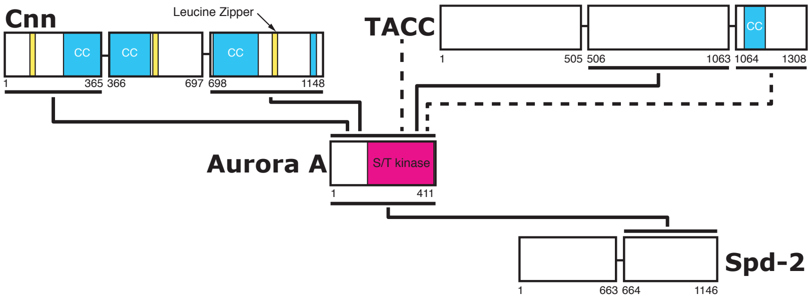

Regulators

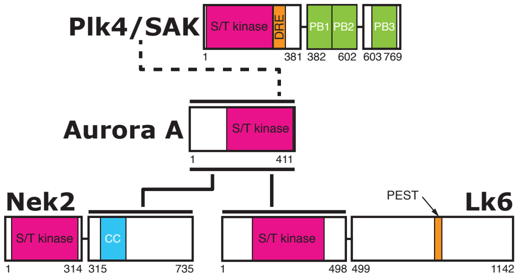

Cnn Interactions

Centriole

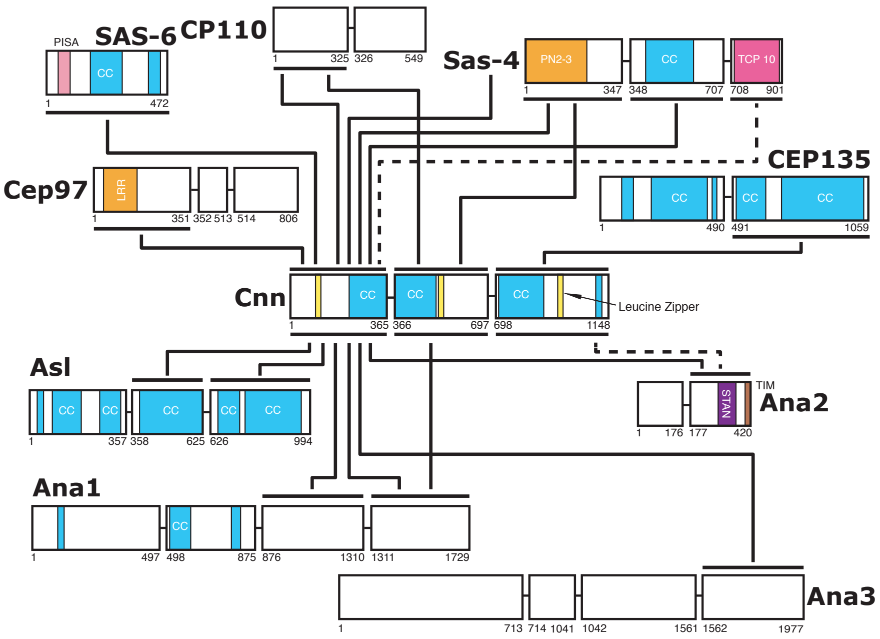

PCM

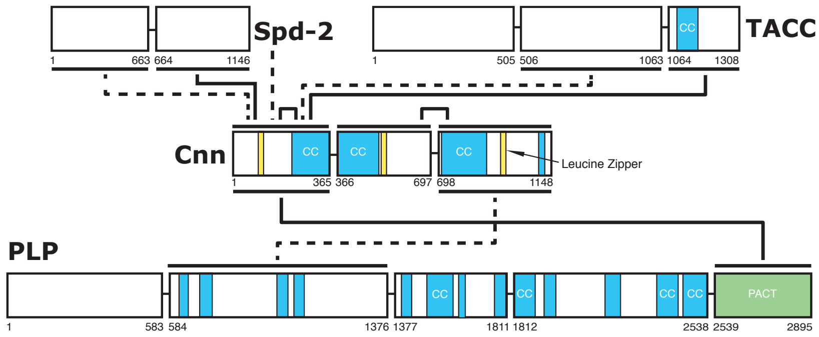

Regulators

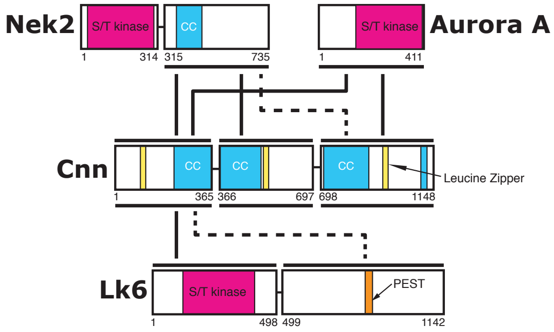

Spd-2 Interactions

Centriole

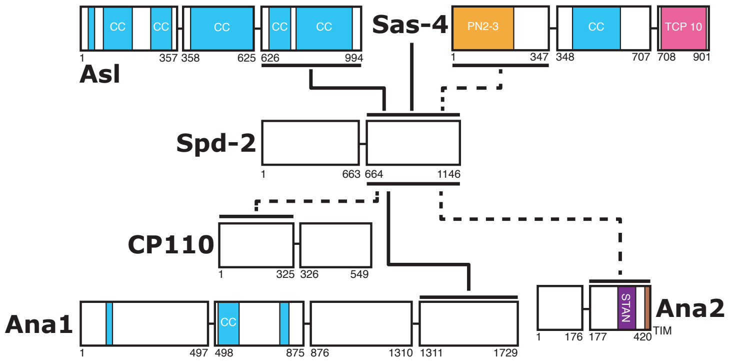

PCM

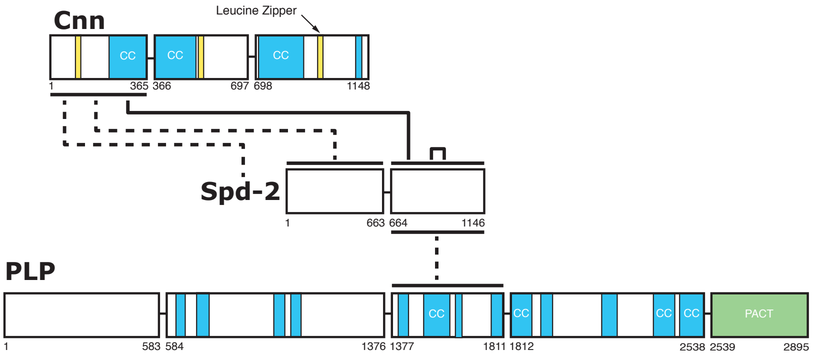

Regulators

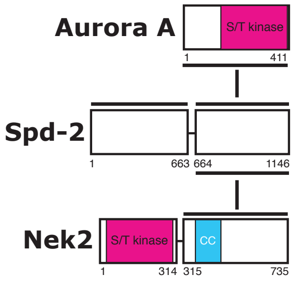

TACC Interactions

Centriole

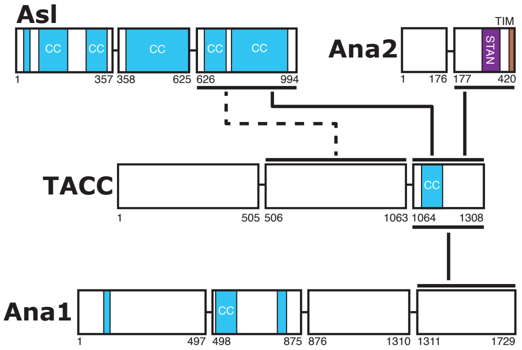

PCM

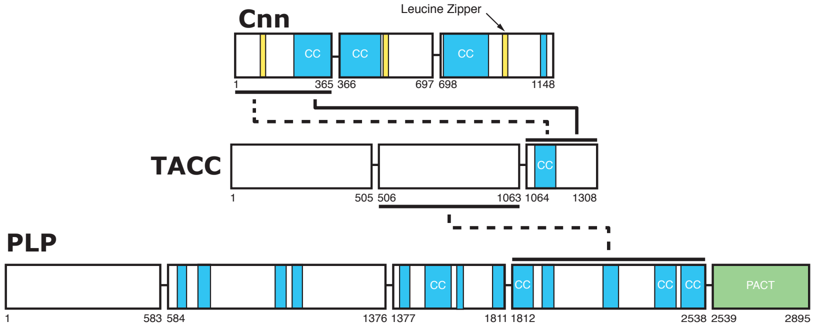

Regulators

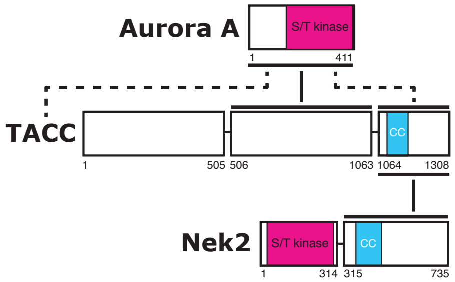

Lk6 Interactions

Centriole

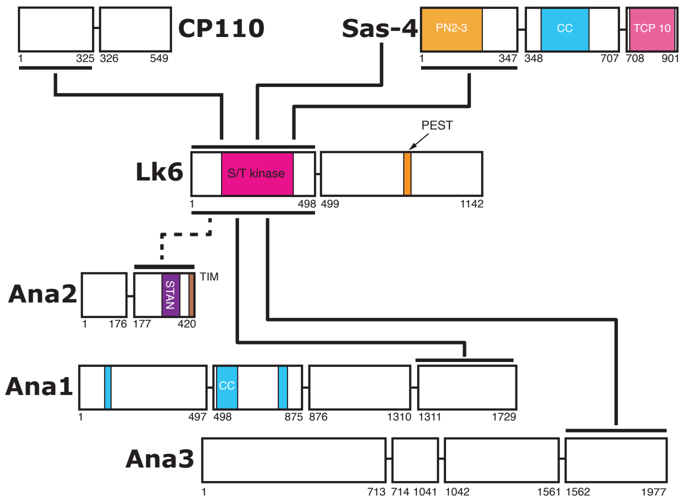

PCM

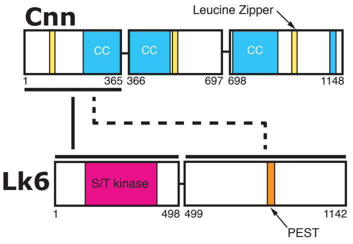

Regulators

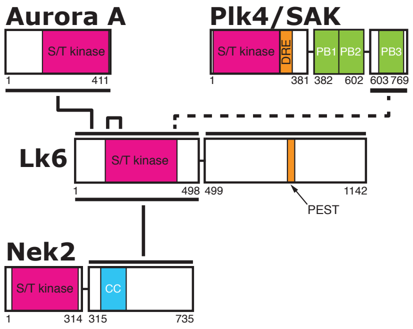

Nek2 Interactions

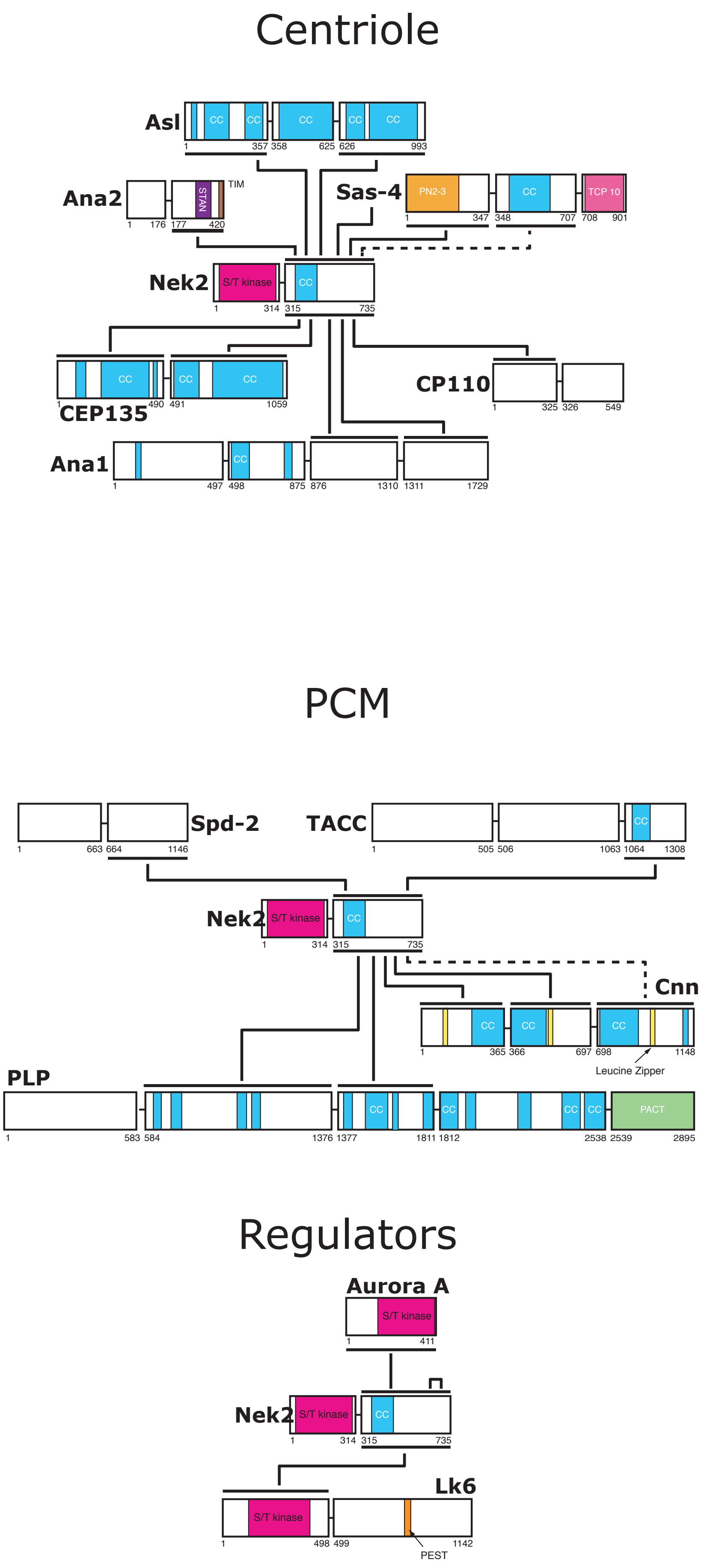

Supplement: Supplementary Data 3 — All Interaction schematic A graphical representation of all of the interactions identified in the Y2H screen at the highest stringency using the same criteria as in the Supplementary Data 2. Interactions with centriole, PCM and regulator proteins are shown separately for each protein. Interactions scored as 2 or 3 are shown with solid lines. Interactions scored a 1 are shown as dashed lines. Lines terminating at the name of the protein indicate an interaction with the full-length protein. Numbers under schematics are amino acid number. Horizontal lines within schematics indicate the locations where proteins were subdivided [file ncomms12476-s4.pdf]
